# Investigating the N-terminal linker histone H1 subtypes as substrates for JmjC lysine demethylases

**DOI:** 10.1039/d5cb00083a

**Published:** 2025-07-28

**Authors:** Vildan A. Türkmen, Anthony Tumber, Eidarus Salah, Samanpreet Kaur, Christopher J. Schofield, Jasmin Mecinović

**Affiliations:** a Department of Physics, Chemistry and Pharmacy, University of Southern Denmark, Campusvej 55 5230 Odense Denmark mecinovic@sdu.dk; b Department of Chemistry and the Ineos Oxford Institute for Antimicrobial Research, Chemistry Research Laboratory, University of Oxford 12 Mansfield Road OX1 3TA Oxford UK christopher.schofield@chem.ox.ac.uk

## Abstract

Members of the Jumonji C (JmjC) subfamily of non-heme Fe(ii) and 2-oxoglutarate (2OG) dependent *N*^ε^-lysine demethylases have established roles in catalysing demethylation of *N*^ε^-methylated lysine residues in core histones; their roles in accepting linker H1 histones as substrates have been less well explored. We report studies on the H1 substrate specificity of human JmjC lysine demethylases (KDMs), specifically KDM3A-C, KDM4A, KDM4D, KDM4E, KDM5D, and KDM6B, for mono-, di- and trimethylated *N*^ε^-lysine residues in peptide fragments of the N-terminal tail of human linker histone H1 isoforms (H1.2, H1.3, H1.4 and H1.5). The KDM4s, but not the other tested JmjC KDMs, catalysed demethylation of tri- and dimethylated H1 peptide isoforms with activities: KDM4E > KDM4D > KDM4A. The order of substrate preference for KDM4E was H1.2K26me3 > H1.5K26me3 ≈ H1.3K24me3 > H1.2K25me3 ≈ H1.4K25me3. For KDM4D, the most efficient tested substrate was H1.5K26me3. Among the dimethylated H1 peptide isoforms, H1.3K24me2 appeared to be the most efficient KDM4E substrate, with comparable activity to the core histone H3K9me2 substrate. The results demonstrate that JmjC KDM4s can accept the N-terminal H1 tails as substrates, further highlighting the potential for flexibility in substrate and product selectivity of the JmjC KDMs, in particular, within the KDM4 subfamily. Molecular and cellular investigations on JmjC KDM-catalysed H1 demethylation are of molecular and biomedical interest.

## Abstract 2

Biomedically important histone lysine demethylases can catalyse demethylation of *N*^ε^-methylated lysine residues in the N-terminal tails of human linker histone H1 isoforms.

## Graphical abstract

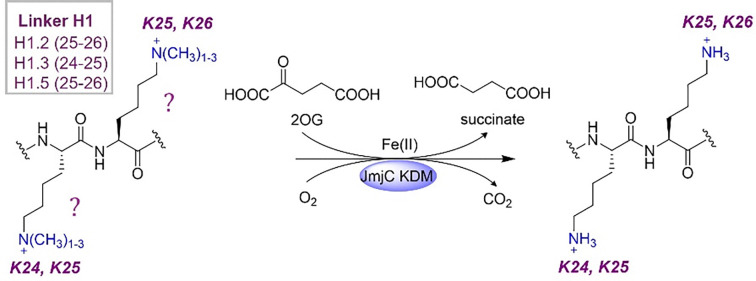

## Introduction

**Fig. 1:**
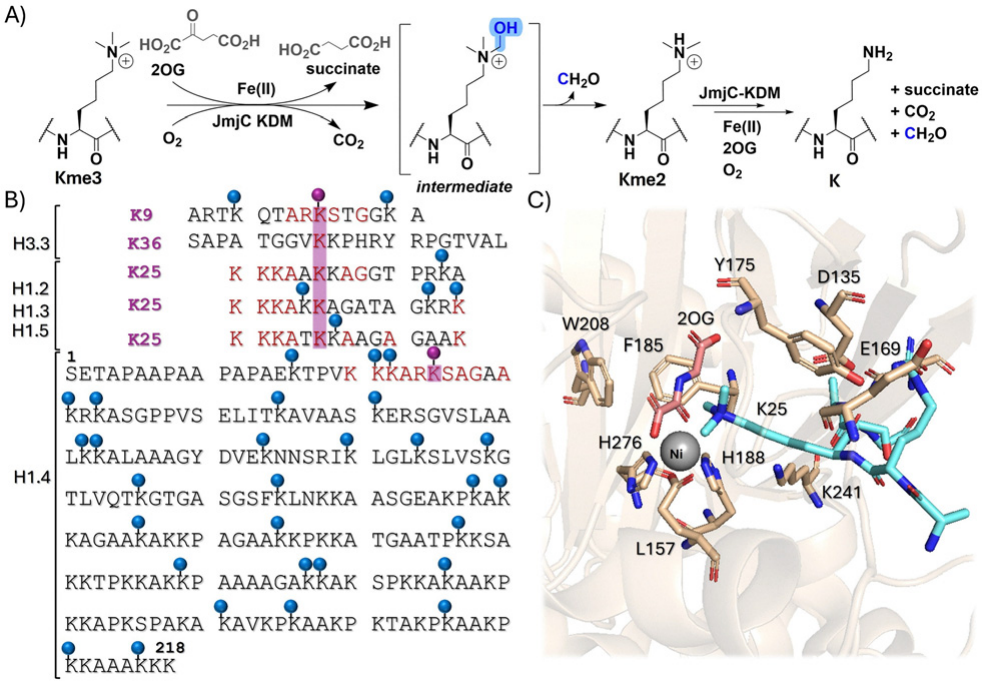
Lysine demethylation of linker histones. (A) JmjC KDM-catalysed demethylation of *N*^ε^-methylated lysine residues. (B) N-terminal sequences of H3, H1.2, H1.3, and H1.5 compared with that of H1.4. Purple dots show Lys-residues of interest for this study. Lys-residues identified as being methylated on H1 subtypes are marked with blue dots. (C) View from a crystal structure of KDM4A bound to a nickel ion (grey, substituting for Fe(ii)), *N*-oxalylglycine (pink, a 2-oxoglutarate analogue inhibitor) and a H1.4K25me3 derived peptide (cyan) (PDB: 6H8P).

*N* ^ε^-Lysine methylations of core histones in chromatin play important roles in the regulation of eukaryotic gene expression.^1,2^ Mono-, di-, and tri-methylations of lysine residues are catalysed by *S*-adenosylmethionine (SAM)-dependent histone lysine methyltransferases (KMTs).^3–5^*N*^ε^-Methylated lysine residues of the N-terminal tail of core histone H3 undergo demethylation at multiple sites, as catalysed by two families of histone lysine demethylases (KDMs): the flavin dependent lysine specific demethylases and the larger family of non-heme Fe(ii) and 2-oxoglutarate (2OG)-dependent JmjC KDMs.^6,7^ The JmjC KDMs catalyse *N*^ε^-methyl demethylation *via* hydroxylation of an *N*^ε^-methyl group, yielding an unstable hemiaminal intermediate, which fragments into the demethylated lysine residue and formaldehyde (Fig. 1A).^8,9^ JmjC KDM dysregulation is associated with multiple diseases, including cancer and neurological disorders,^10,11^ making some KDMs current targets for cancer drug development.^10,12^ The JmjC KDMs primarily target lysine residues on histone and non-histone proteins, with arginine residues also being targets for demethylation.^13–15^ While links between some core histone modifications, notably lysine methylation and acetylation, and chromatin structure and function are established, it is less clear how post-translational modifications (PTMs) on linker histone H1 isoforms affect chromatin.^16–18^

The eleven human H1 isoforms undergo PTMs, including *N*^ε^-lysine methylation.^16,18^ While H1 methylation has been observed, its precise extent and influence on gene expression have been unclear and are the subject of ongoing research.^1,16,18^ Multiple lysine methylation sites have been identified on the N-terminal tail, the globular domain, and the C-terminal tail of H1.^18,19^ Histone 1 isoform 4 (H1.4) is methylated at K25 (H1.4K25) and the H1.4K25 methylation profile is reported to vary across different stages of the cell cycle.^19,20^ Substitution of alanine at position K25 on H1.4 results in reduction in cell proliferation and stabilisation of H1.4 in chromatin compaction.^20^ Murine studies have revealed that KDM4 can catalyse the removal of methylated H1.4K25, thus promoting transcription.^19–21^ Notably, the H1.4K25 residue is conserved among the H1.2, H1.3, and H1.5 linker histone subtypes, raising the question of whether these subtypes undergo JmjC KDM-catalysed demethylation (Fig. 1B).^18,19,21^

All members of the JmjC KDM4 subfamily (KDM4A-E) catalyse demethylation of core histone H3K9me3/2, with an apparent preference for the tri- and di-methylated states.^22,23^ KDM4-catalysed demethylation of H3K36me3 also occurs, but is specific to KDM4A-C, which possesses additional Tudor and PHD domains compared to KDM4D/E.^22^ Notably, H1.4 (H1.4_23–26_) contains a similar ARKS sequence motif to that present in H3K9_7–10_ and H3K27_25–28_, which is recognised by the reader chromodomain of Chromodomain Y-like 2 (CDL2) and HP1.^21,24,25^*N*^ε^-Methylated H1.4K25 is reported to be a substrate for JmjC KDM4 subfamily proteins, including KDM4A, KDM4D and KDM4E, which demethylate H1.4K25me3 with varying efficiencies.^26^ Here, we report studies on the substrate selectivity of human JmjC KDMs for demethylation of somatic human H1 isoforms, focusing on the methylated H1.4K25 equivalent and adjacent lysine residues present on the N-terminal tail in each H1 isoform.

## Results and discussion

**Fig. 2:**
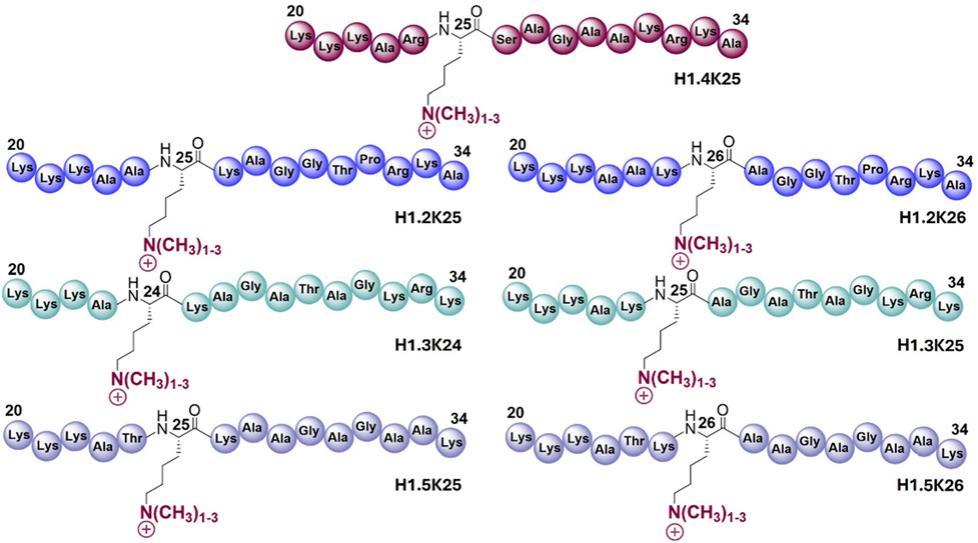
The N-terminal tail H1 substrate specificity of KDMs. Methylated lysine residues were incorporated into H1.4K25, H1.2K25, H1.2K26, H1.3K24, H1.3K25, H1.5K25, and H1.5K26 isoform peptides.

We aimed to explore the substrate specificity of KDM-catalysed demethylation of methylated lysine residues in H1.2–H1.5 peptides possessing mono-, di-, and tri-methylated lysine residues 24–26 corresponding to equivalent and adjacent positions (K24, K25 and K26) to the established H1.4K25 methylation site. Structural analyses have revealed that H1.4K25me3 is likely bound within the active site cleft of KDM4A through its backbone residues, primarily *via* hydrogen bonding interactions in a similar manner to H3K9me3/2 binding (Fig. 1C).^26^ The tri-methylated lysine residue substrate is positioned near Fe(ii) ion, which is essential for the oxidative demethylation (Fig. 1C).^26^ We therefore initially tested *N*^ε^-tri-methylated 20-mer and 15-mer H1.4 peptides using previously optimised conditions to determine an appropriate sequence for detailed enzyme assays.^26^ Consistent results were obtained with the two peptide lengths, and hence further work proceeded with 15-mer H1.4 peptide fragments (Fig. S1). A set of 15-mer peptides of H1 isoforms (residues 20–34), H1.2, H1.3, H1.4 and H1.5, was synthesised, with mono-, di-, and tri-methylated lysine residues at positions 24–26 (Fig. 2 and Table S1). All peptides were synthesised using automated Fmoc-based solid-phase peptide synthesis (SPPS) on a Rink amide resin and purified by preparative HPLC (Table S1; H3K9me3 and H3K36me3/2 were obtained commercially).

**Fig. 3:**
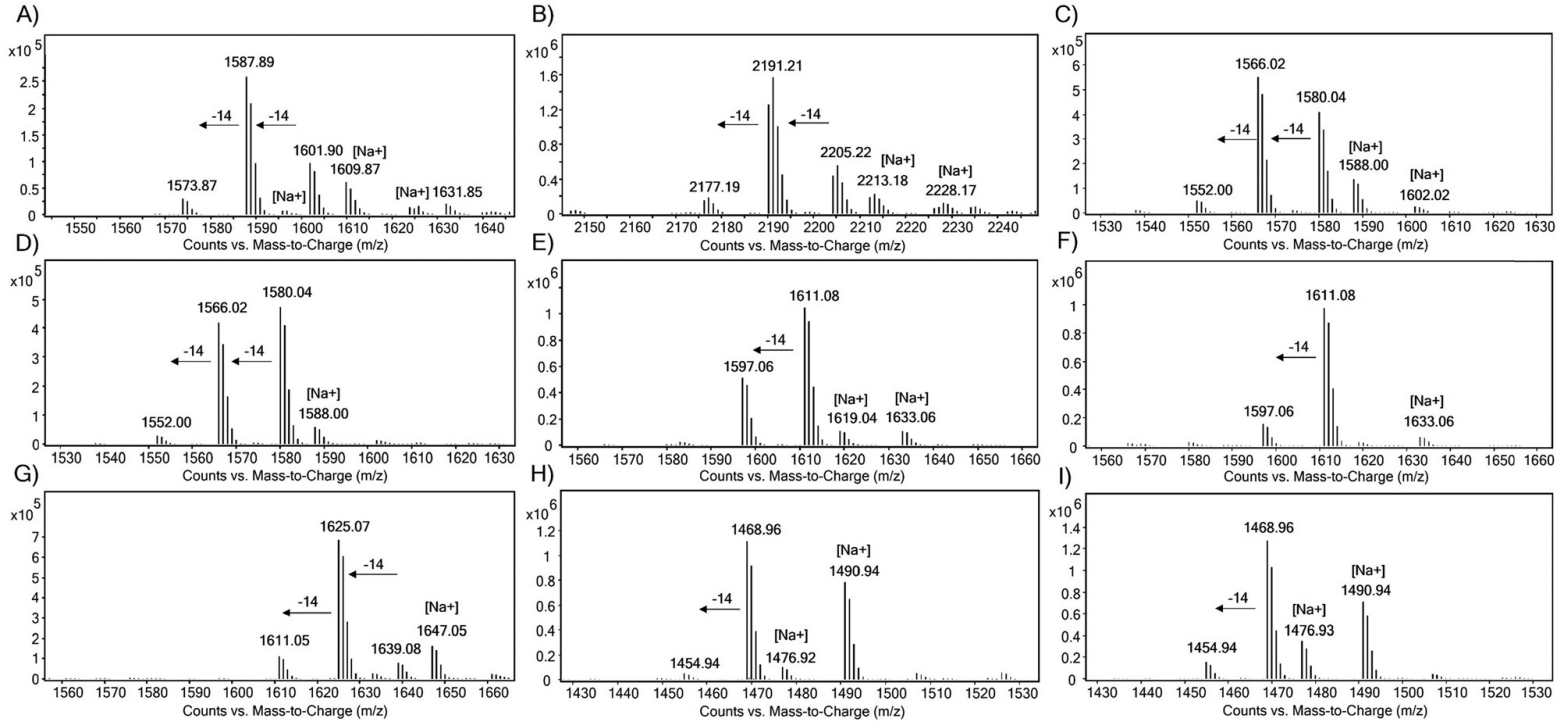
LC-MS spectra showing the KDM4A-catalysed lysine demethylation of (A) H3K9me3, (B) H3K36me3, (C) H1.2K25me3, (D) H1.2K26me3, (E) H1.3K24me3, (F) H1.3K25me3, (G) H1.4K25me3, (H) H1.5K25me3, and (I) H1.5K26me3.

**Fig. 4:**
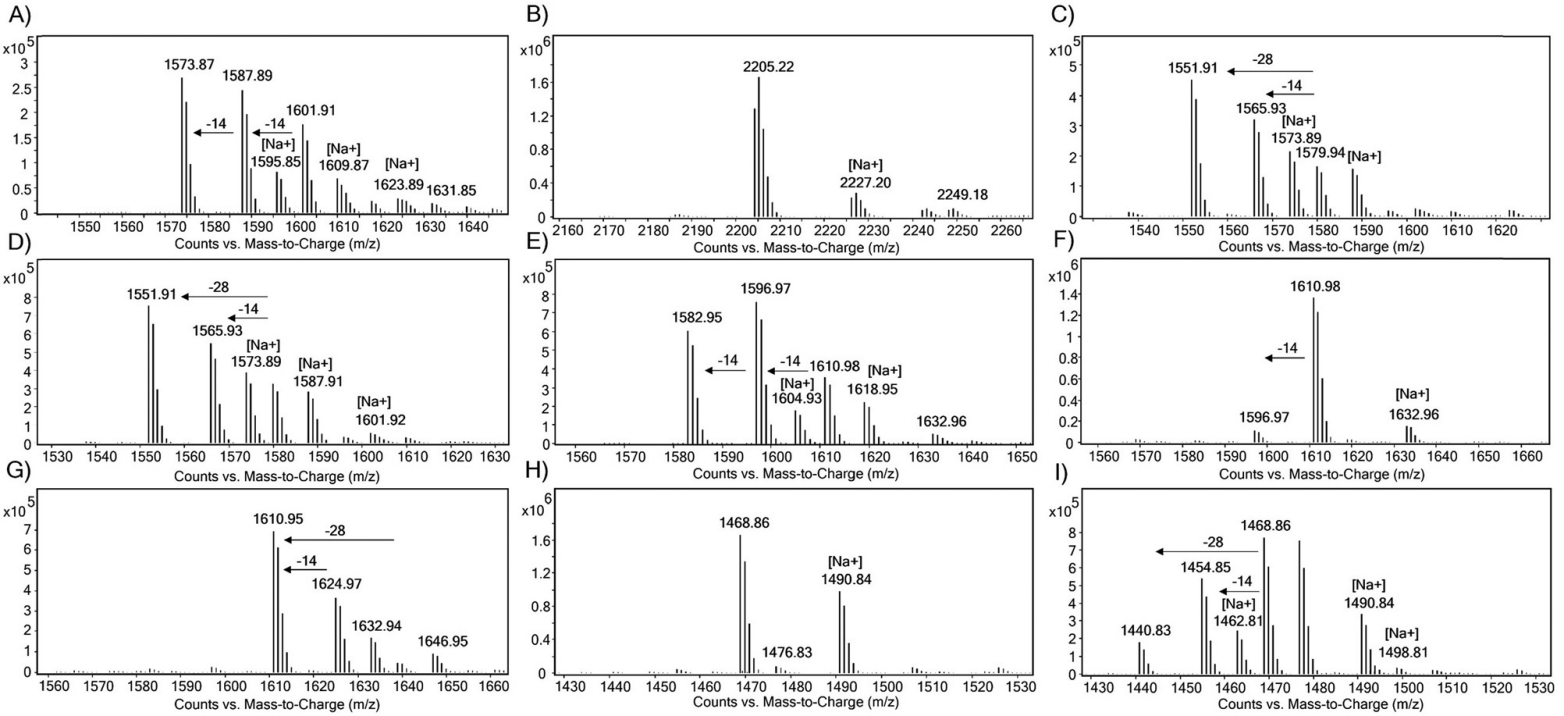
LC-MS spectra for KDM4D-catalysed lysine demethylations of (A) H3K9me3, (B) H3K36me3, (C) H1.2K25me3, (D) H1.2K26me3, (E) H1.3K24me3, (F) H1.3K25me3, (G) H1.4K25me3, (H) H1.5K25me3, and (I) H1.5K26me3.

**Fig. 5:**
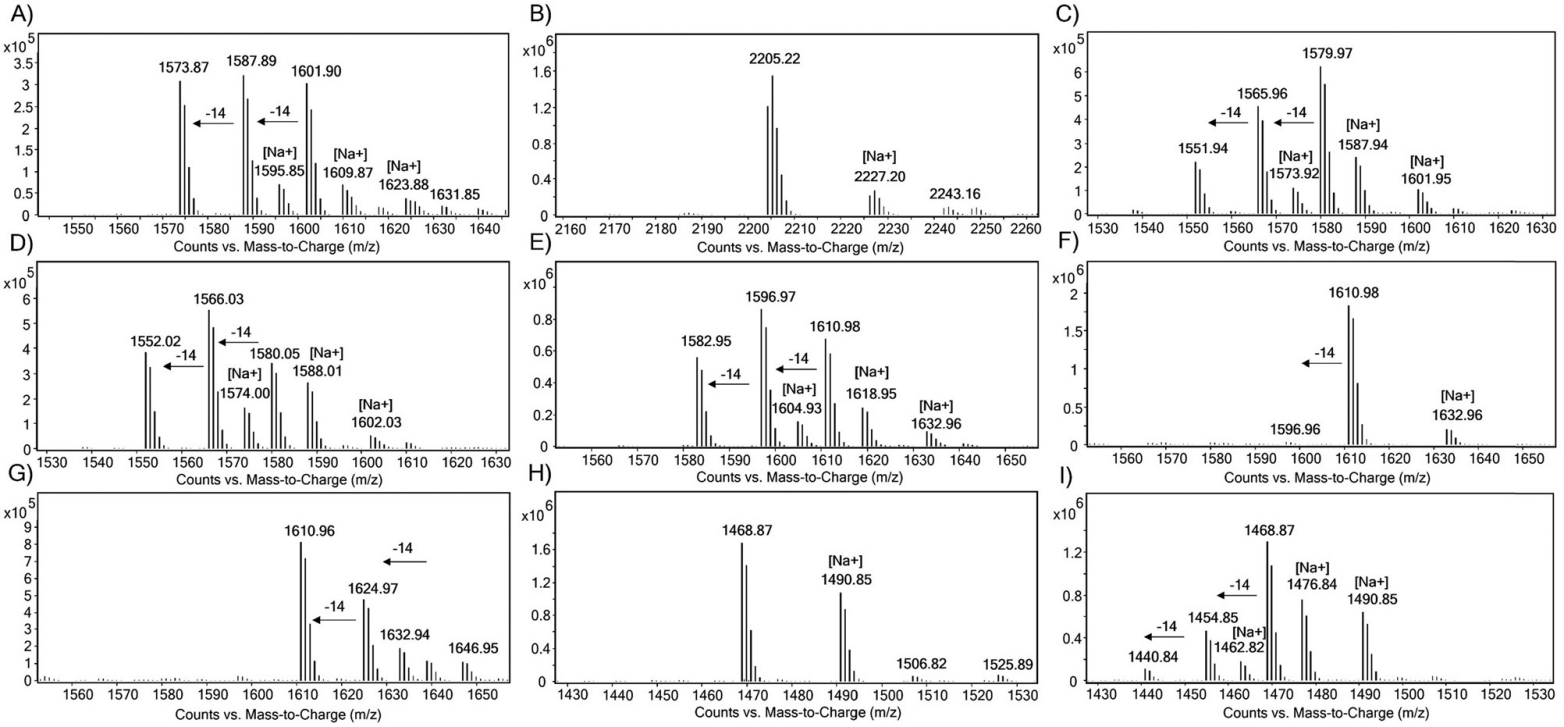
LC-MS spectra showing the KDM4E-catalysed lysine demethylation of (A) H3K9me3, (B) H3K36me3, (C) H1.2K25me3, (D) H1.2K26me3, (E) H1.3K24me3, (F) H1.3K25me3, (G) H1.4K25me3, (H) H1.5K25me3, and (I) H1.5K26me3.

The substrate specificity of JmjC KDMs for H1 was examined using liquid chromatography coupled to mass spectrometry (LC-MS) assays. The H1 peptides (10 μM) were incubated with the recombinant KDM (2 μM) in HEPES buffer at pH 7.5 at room temperature for 2 h, in the presence of ferrous(ii) ammonium sulfate (FAS, 10 μM), sodium ascorbate (LAA, 100 μM) and 2OG (20 μM), and then quenched with formic acid. *N*^ε^-Methylated H3K9 and H3K36 peptides were used as positive controls (Fig. 3–5 and Fig. S2–S6).^26^ Under the standard conditions, KDM3A-C, KDM5D and KDM6B did not exhibit any activity with the tri-methylated H1 peptides (Fig. S2–S6). In contrast, KDM4A-, KDM4D- and KDM4E-catalysed demethylation was observed for most of the tested tri-methylated lysine residues of H1 peptides, with varying levels of efficiency (Fig. 3–5, Fig. S7 and Table 1).

**Table 1:** Summary of activity results for recombinant human JmjC KDMs on *N*^ε^-lysine methylated H1_20–34_ peptides under standard conditions (2 μM KDM, 10 μM H1/H3 peptide, 10 μM FAS, 100 μM LAA, 20 μM 2OG, 2 h, room temperature). Red dots indicate no activity under standard conditions; black dots indicate low-level demethylation (<10% conversion); yellow dots indicate moderate demethylation (<50% conversion); green dots indicate good demethylation (>50% conversion); M (mono-), D (di-) and T (tri-) represent the initial methylation states for the H1/H3 peptide substrates

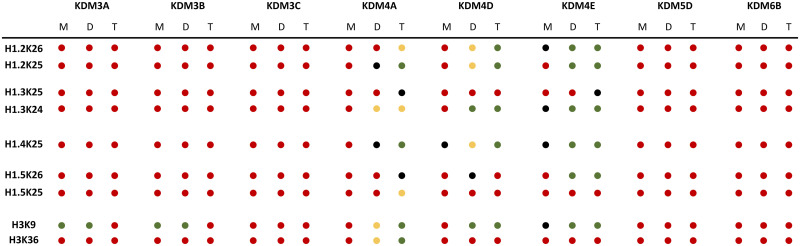

In the presence of KDM4A, the positive control peptides H3K9me3 and H3K36me3 were converted predominantly to di-methylated products under standard conditions, yielding ∼53% di- and >10% mono-methylated products for H3K9me3 and ∼68% di- and >10% mono-methylated products for H3K36me3 (Fig. 3A and B). We did not observe any evidence of hydroxylation, as observed with KDM4D and KDM4E for Arg20 of H2a, in any of the substrates tested in our work.^27^ As reported,^26^ we observed H1.4K25me3 to be a KDM4A substrate, as demonstrated by the ∼87% substrate turnover (Fig. 3G). The H1.2 isoform was observed to be a good KDM4A substrate with a conversion of ∼51% and ∼45% at the K26me3 and K25me3 sites, respectively (Fig. 3C and D). Moreover, the H1.3 isoform was observed to be less efficiently demethylated by KDM4A at both sites, with only ∼36% (K24me3) and <10% (K25me3) conversion (Fig. 3E and F). H1.5 underwent poor KDM4A-catalysed demethylation when H1.5K25 and H1.5K26 were tri-methylated, giving <10% of the demethylated lysine residue (Fig. 3H and I). Overall, these findings suggest that KDM4A catalyses demethylation of the H1 isoform with the following order of efficiency: H1.4K25me3 > H1.2K25me3 ≈ H1.2K26me3 > H1.3K24me3 > H1.5K26me3 ≈ H1.3K25me3 > H1.5K25me3 (Fig. S7).

We next explored the H1 substrate specificity of KDM4D (Fig. 4). In control assays, KDM4D exhibited ∼53% demethylation for H3K9me3, resulting in the formation of ∼35% di- and ∼18% mono-methylated products (Fig. 4A). No demethylation activity was observed for H3K36me3 in the presence of KDM4D, consistent with reports indicating that H3K36me3 is not a KDM4D substrate (Fig. 4B).^28^ With KDM4D, we observed substantial conversion of the tri-methylated H1.2 and H1.3 peptides, in particular, at lysine positions H1.2K26me3 (∼70% conversion) and H1.3K24me3 (∼68% conversion), primarily yielding di- (∼30%) and mono-methylated (∼40%) products (Fig. 4D and E). No activity was observed for H1.3K25me3 and H1.5K25me3 with KDM4D under the tested conditions (Fig. 4F and H). In contrast, H1.4K25me3 was an efficient KDM4D substrate (∼92% conversion), giving ∼30% di- and ∼62% mono-methylated products (Fig. 4G). These results show that KDM4D catalyses demethylation of H1 isoform derived peptides with the following order of efficiency: H1.4K25me3 > H1.2K25me3 ≈ H1.2K26me3 ≈ H1.3K24me3 > H1.5K26me3 > H1.3K25me3 ≈ H1.5K25me3 (<5% di-methylation) (Fig. S7).

With KDM4E, H3K9me3 was efficiently demethylated, yielding ∼25% di- and ∼29% mono-methylated peptide products (Fig. 5A). As reported, the H3K36me3 peptide was not a KDM4E substrate (Fig. 5B).^28^ With KDM4E, H1.2K25me3 and H1.2K26me3 peptides exhibited ∼80% substrate conversion, predominantly producing mono- (∼30%) and di-methylated (∼50%) products (Fig. 5C and D). Like KDM4D, KDM4E catalysed efficient demethylation of H1.3K24me3 (∼80%), producing ∼42% di- and ∼38% mono-methylated products, whereas H1.3K25me3 was observed to be a poor KDM4E substrate (<10% conversion) (Fig. 5E and F). With H1.4K25me3, KDM4E also catalysed efficient demethylation (∼95% conversion), showing ∼28% di- and ∼67% mono-methylated products (Fig. 5G). With KDM4E, H1.5K26me3 underwent conversion to yield ∼40% of di- and ∼16% of mono-methylated products, but H1.5K25me3 was not a substrate for KDM4E (Fig. 5H and I). These results show that KDM4E demethylates H1 isoforms with a similar order of efficiency to KDM4D, that is: H1.4K25me3 > H1.2K25me3 ≈ H1.2K26me3 ≈ H1.3K24me3 > H1.5K26me3 > H1.3K25me3 ≈ H1.5K25me3 (Fig. S7).

We then explored whether di-methylated H1 peptides serve as substrates for human JmjC KDMs; demethylation activity was observed only in the presence of the JmjC KDM4 subfamily enzymes (Table 1 and Fig. S8–S15). With KDM4A, the H3K9me2 and H3K36me2 control peptides underwent ∼36% conversion (Fig. S11A and B). The H1.3K24me2 peptide underwent ∼23% conversion (Fig. S11E), whereas the H1.4K25me2 peptide was converted to a similar extent as H1.3K24me2 (∼20%) (Fig. S11G). The other tested di-methylated H1 isoforms appear to be unmodified by KDM4A treatment within detection limits (Fig. S11). With KDM4D, the H3K9me2 peptide underwent ∼58% demethylation (Fig. S12A); H3K36me2 did not undergo demethylation, as reported (Fig. S12B).^28,29^ With H1.2K25me2 and H1.2K26me2, KDM4D catalysed 20–35% conversion to the mono-methylation state (Fig. S12C and D) and H1.4K25me2 underwent >40% conversion to a mono-methylated state (Fig. S12G). The H1.3K24me2 peptide showed a similar demethylation pattern as the H3K9me2 positive control, with mono-methylated lysine as the predominant product (∼53% conversion) and a smaller extent of non-methylated product (∼8%) (Fig. S12E). This was the highest conversion level observed amongst the tested dimethylated H1 peptides (Fig. S12). With KDM4E, the H3K9me2, H1.2K25me2, and H1.2K26me2 peptides underwent similar levels of conversion to principally mono-methylated products (∼64%) (Fig. S13C and D), while H1.3K24me2 showed predominant conversion to the unmethylated lysine product (∼60%) (Fig. S13E). With KDM4E, H1.4K25me2 showed ∼50% demethylation, while H1.5K26me2 exhibited >40% demethylation (Fig. S13G and I). These findings further showed that the KDM4 subfamily members have distinct substrate preferences and demethylation efficiencies, with KDM4E manifesting the highest activity, particularly for H1.2K25me2, H1.2K26me2 and H1.3K24me2 amongst the tested di-methylated peptides, an observation consistent with the results reported for the tri-methylated peptides (Fig. 5 and Fig. S13).

We also explored whether mono-methylated H1 peptides are substrates for the JmjC KDMs, but none exhibited activity within the detection limits, at least under the tested conditions (Table 1 and Fig. S16–S23). A lack of activity is consistent across various monomethylated histone peptides, including the H3K9me peptide, except for KDM3A and KDM3B, which, as anticipated, exhibited excellent activity with H3K9me (Fig. S16 and S17).

The LC-MS assay used for screening is not suitable for quantitative kinetic analysis, and hence we conducted Michaelis–Menten type kinetic analyses for KDM4D and KDM4E, using a reported fluorescence-based formaldehyde dehydrogenase (FDH)-coupled demethylation assay (Fig. S25 and Table 2), in which FDH-catalysed oxidation of formaldehyde is coupled to NADH formation, which is measured by fluorescence.^29,30^ Kinetic parameters were determined by recording the initial reaction rates of NADH production at varying histone peptide concentrations (Fig. S24 and S25). The overall catalytic efficiencies (as measured by *k*_cat_/*K*_M_) gave the following rank orders for KDM4D: H1.5K26me3 (0.192 μM^−1^ min^−1^) > H1.3K24me3 (0.066 μM^−1^ min^−1^) ≈ H1.2K26me3 (0.063 μM^−1^ min^−1^) > H1.4K25me3 (0.044 μM^−1^ min^−1^). For KDM4E, the kinetic rank order was as follows: H3K9me3 (0.585 μM^−1^ min^−1^) > H1.2K26me3 (0.360 μM^−1^ min^−1^) > H1.5K26me3 (0.324 μM^−1^ min^−1^) ≈ H1.3K24me3 (0.310 μM^−1^ min^−1^) > H1.2K25me3 (0.247 μM^−1^ min^−1^) ≈ H1.4K25me3 (0.226 μM^−1^ min^−1^). These rank orders differ somewhat from the initial screening results. However, given the complexities of 2OG oxygenase catalysis and the relatively low catalytic efficiencies observed, such apparent discrepancies are not unexpected.^28^ It is also noteworthy that, in some instances, increased *K*_M_ values correlate with elevated *k*_cat_ values, resulting in similar *k*_cat_/*K*_M_ ratios, particularly for H1.4K25me3 with KDM4D and H1.2K25me3 with KDM4E. These observations underscore the mechanistic and kinetic complexity inherent in JmjC KDM catalysis, indicating that a simple mechanistic interpretation of *K*_M_ and *k*_cat_ values is problematic, including *K*_M_ values that do not necessarily reflect *K*_D_ values.

Kinetic parameters for demethylation of *N*^ε^-trimethylated H1 peptides by KDM4D and KDM4E (400 nM) measured using a formaldehyde dehydrogenase-coupled demethylation assay. H1 peptide (1–50 μM), FAS (20 μM), LAA (200 μM), 2OG (200 μM), NAD^+^ (500 μM), KDM4 enzyme (0.8 μM) and FDH (2.0 μM) in HEPES buffer at pH 7.5. Each value is shown as mean ± SEM (*n* = 3)

**Table:** 

KDM4D	*K*_M_/μM	*k*_cat_/min^−1^	*k*_cat_/*K*_M_ μM^−1^ min^−1^
H1.2K26me3	4.60 ± 0.41	0.29 ± 0.01	0.063
H1.3K24me3	5.93 ± 0.69	0.39 ± 0.02	0.066
H1.4K25me3	60.0 ± 24.3	2.66 ± 0.91	0.044
H1.5K26me3	7.51 ± 0.77	1.44 ± 0.08	0.192

**Table:** 

KDM4E	*K*_M_/μM	*k*_cat_/min^−1^	*k*_cat_/*K*_M_ μM^−1^ min^−1^
H3K9me3	6.72 ± 1.51	3.93 ± 0.19	0.585
H1.2K25me3	18.5 ± 6.87	4.60 ± 1.31	0.247
H1.2K26me3	5.22 ± 0.54	1.88 ± 0.09	0.360
H1.3K24me3	6.45 ± 1.51	2.00 ± 0.25	0.310
H1.4K25me3	11.5 ± 3.19	2.61 ± 0.47	0.226
H1.5K26me3	4.04 ± 0.51	1.31 ± 0.08	0.324

**Fig. 6:**
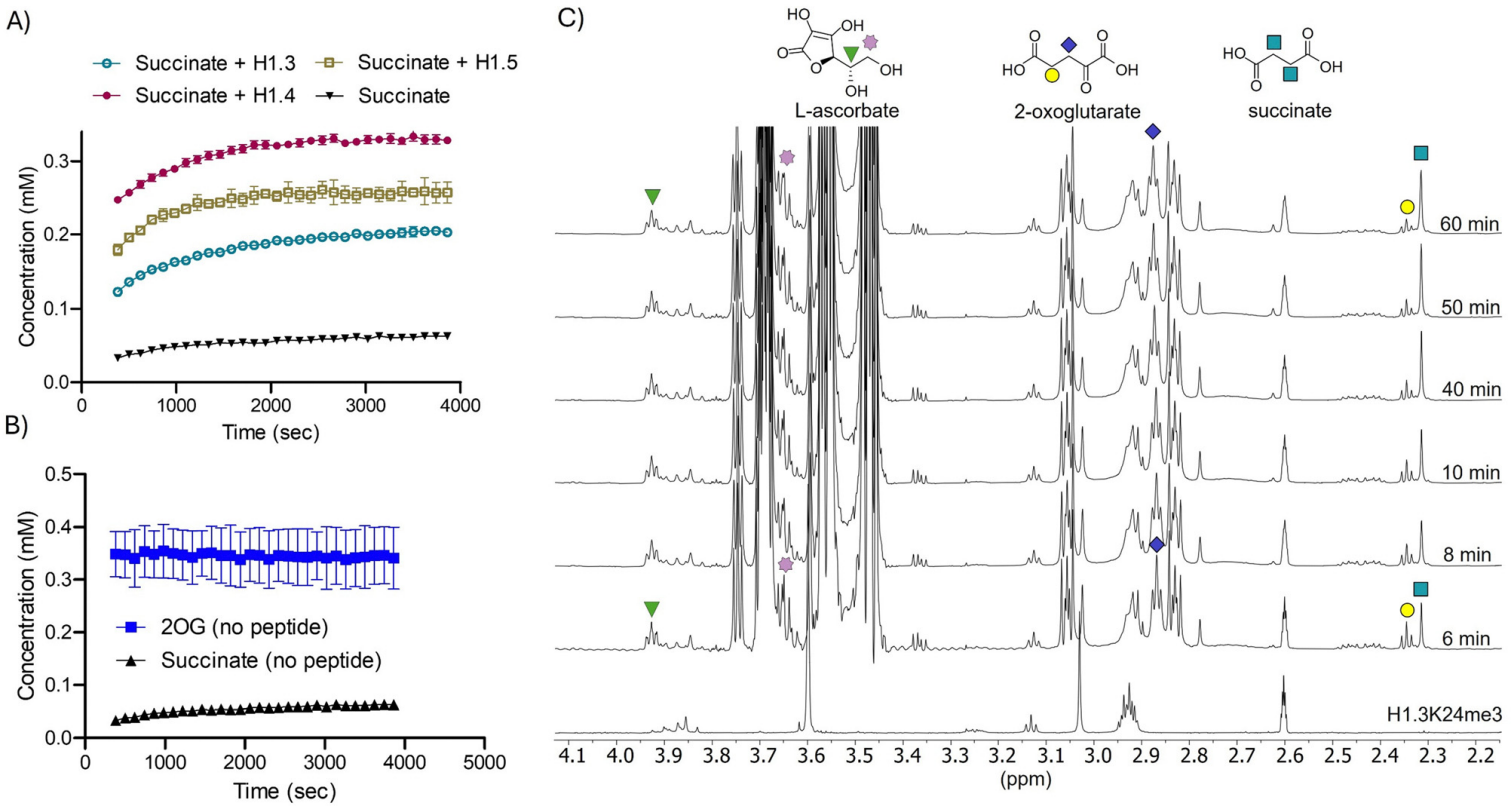
Time-course ^1^H NMR analysis for reactions of KDM4D with H1 peptides. (A) KDM4D-catalysed succinate production for H1.3K24me3 (turquoise), H1.4K25me3 (raspberry), and H1.5K26me3 (khaki) compared to that without peptides (black). (B) KDM4D in the presence of 2OG without the H1 peptide; note that only low-level succinate production is observed. (C) Production of succinate measured for KDM4D/H1.3K24me3 over time, as evidenced by increasing integration of the singlet at *δ*^1^H 2.31 ppm (corresponding to the succinate methylenes). Assay conditions are described in the Experimental details section.

^1^H NMR analyses with KDM4D and H1.3K24me3, H1.4K25me3 or H1.5K26me3 monitoring succinate production arising from 2OG oxidation (Fig. 6 and Fig. S26–S28) provided clear evidence for oxidation of all three H1 peptides, as demonstrated by the increasing levels of succinate (*δ*^1^H 2.31 ppm) and decreasing 2OG resonances (*δ* 2.85 ppm and 2.35 ppm) (Fig. 6A). With this assay, H1.3K24me3 and H1.5K26me3 showed slightly slower succinate production compared to H1.4K25me3. A negative control without the H1 peptide using KDM4D exhibited only a low level of succinate formation, likely due to substrate uncoupled 2OG turnover by KDM4D and/or non-enzymatic oxidation (Fig. 6B, C and Fig. S26).^31^

## Conclusions

Both core and linker histones undergo lysine methylations at multiple sites, some of which are reported to regulate the chromatin structure and function.^32,33^*In vivo* studies on H1 PTMs are challenging due to sequence similarities and because H1 isoforms possess numerous lysine and other residues with potential for PTMs, complicating antibody and MS based analyses. The full extent of PTMs on H1 isoforms is thus unclear, with only limited available knowledge of the enzymes responsible for installing and, potentially, removing H1 methyl-lysine marks. Although our work involved studies with histone H1 derived peptides and purified recombinant catalytic domains of JmjC KDMs, the results provide a useful molecular basis for functional assignment of enzyme–substrate combinations *in vivo*.

Building on previous findings that H1.4 is a substrate for KDM4 JmjC subfamily enzymes,^26^ the H1 screening studies described here reveal the potential for KDM4-catalysed H1 demethylation. KDM4E, followed by KDM4D, demonstrated the highest demethylation activity of the H1 peptides, particularly for the H1.2K25me3/2, H1.2K26me3/2 and H1.3K24me3/2 peptides. Demethylation activities were not observed with the other tested JmjC KDMs, that is, KDM3A-C, KDM5D and KDM6B (note that it is unclear if KDM3C possesses demethylation activity), consistent with a previous report on the lack of demethylation with KDM5D and KDM6B for H1.4.^26^

In general, the trimethylated H1 substrates appeared more active than the di- or mono-methylated substrates, though it is important to note that JmjC KDM activities are condition- and sequence-context dependent.^13,27–30,34^ One striking observation from our results is that KDM4E displays similar activity for H1.3K24me2 compared to its established H3K9me2 substrate.^28–30^

The results indicate that KDM4D and KDM4E may exhibit different selectivities for H1 substrates. Importantly, more detailed kinetic and selectivity assays, including with more biologically representative substrates (*i.e.*, both nucleosomes and chromatin), are required to validate this observation. Notably, H1.3K25me3 and H1.5K25me3 were not demethylated by any member of the KDM4 subfamily, suggesting that not all *N*^ε^-lysine methylated H1 proteins are KDM4 substrates. It is also important to note that JmjC KDMs not included in our study may act on H1 isoforms, and those found to be inactive in our study may require additional H1 PTMs (or other chromatin components) for efficient activity, as is the case for some JmjC KDM/H3 substrate pairs.^35–37^

Previous studies have revealed different selectivities within the KDM4A-E subfamily, including that KDM4A-C accept both H3K9me3/2 and H3K36me3/2 but do not accept H3K36me3/2.^28,29^ In contrast, KDM4D/E appear to be more efficient N-terminal arginine demethylases than KDM4A-C, and KDM4E can catalyse arginine C-4 hydroxylation (*e.g.*, H2a R20).^27^ Interestingly, amongst the tested enzymes, KDM4D and KDM4E exhibited clear demethylase activity for some of the di-methylated H1 peptides; notably, KDM4E displayed activity with H1.3K24me2 comparable to H3K9me2. The results thus further highlight the potential for flexibility in substrate and product selectivities of the JmjC KDMs, in particular for the KDM4 subfamily, and especially for KDM4E. Although further biochemical and cellular studies are needed to comprehensively identify KDMs acting on H1 isoforms, our overall findings support the potential for JmjC KDM-catalysed modifications of H1 isoforms. In particular, our findings support the proposal that KDM4-catalysed demethylation of the N-terminal region of H1 may contribute to epigenetic regulation,^26^ indicating that further exploration of H1 demethylation in cellular and *in vivo* studies is warranted. Given that the methylation H1.4K25 varies across different stages of the cell cycle,^18,19^ exploring potential roles of KDM4-catalysed demethylation at H1.4K25 is of particular interest.

## Experimental details

### Protein production

The catalytic domains of the JmjC KDMs used were recombinantly produced as reported in either *Escherichia coli* (KDM4A_M1-L359_,^38^ KDM4D_M1-L359_,^39^ KDM4E_M1-R336_,^30^ KDM6B_D1141–E1590_^40^ and KDM3C_L2157-L2500_) or Sf9 cells (KDM3A_T515–S1317_, KDM3B_882–1761_, and KDM5D_M1-D775_) as N-terminally His6-tagged proteins. Proteins were purified by Ni-affinity chromatography followed by size exclusion chromatography to a highly purified state (>90% by SDS-PAGE analysis).^30,38–40^ To produce proteins, DNA sequences encoding for KDM3A, KDM3B and KDM5D were inserted into the pFB-LIC vector, containing a TEV-protease cleavable N-terminal 6x-histidine tag *via* ligation independent cloning (LIC). The resultant plasmids were transformed into baculovirus and Sf9 cells (2 × 106 cells L^−1^) which were infected with the virus stock. The cells were grown at 27 °C and 90 rpm for 70 h. Cells were then harvested and suspended in 100 mL of lysis buffer (50 mM HEPES-NaOH, pH 7.4, 500 mM NaCl, 20 mM imidazole, 0.5 mM Tris (2-carboxyethyl)phosphine (TCEP), 5% (v/v) glycerol). The cells were lysed by sonicating on ice using a 13-mm probe VCX 500 (3 min, 35% amplitude, 5 s on, and 10 s off.). The supernatant was loaded onto a Ni-NTA gravity column, washed with 10 column volume of lysis buffer and eluted using the elution buffer (50 mM HEPES-NaOH, pH 7.4, 500 mM NaCl, 300 mM imidazole, 0.5 mM TCEP, 5% (v/v) glycerol). The eluted protein was further purified using S200 gel filtration column chromatography (50 mM HEPES-NaOH, pH 7.4, 150 mM NaCl, 0.5 mM TCEP and 5% glycerol). Protein purity was analysed by SDS-PAGE and further confirmed by mass spectrometry. Selected fractions were pooled, concentrated and stored at −80 °C.

### Peptide synthesis

The 15-mer H1_20–34_ peptides were assembled manually using the Rink amide resin until position A27 of H1.2, A26 of H1.3, S26 of H1.4, and A27 of H1.5. The Fmoc-protected *N*^ε^-methylated lysine residues were coupled manually with a molar equivalent ratio of 2 : 2 : 4 for Fmoc protected amino acid:hexafluorophosphate azabenzotriazole tetramethyl uronium (HATU):*N*,*N*-diisopropylethylamine (DIPEA) and were reacted overnight at room temperature. The remainder of the sequences were assembled using PurePep^TM^ Chorus (Gyros Protein Technologies). Couplings were performed using a molar ratio of 0.2 : 0.25 : 0.25 for Fmoc-protected amino acid:diisopropylcarbodiimide (DIC):ethyl cyano(hydroxyimino)acetate (OxymaPure) at 90 °C for 2 min, with double-couplings and deprotected steps being employed, utilizing 20% (v/v) piperidine for 2 min at 90 °C for the mono- and trimethylated H1 peptides. For the dimethylated peptides, double couplings at 60 °C for 20 min were employed followed by Fmoc deprotection with 20% (v/v) piperidine for 4 min at 60 °C. Standard cleavage conditions from resin were employed using 2.5% (v/v) triisopropyl silane (TIPS) and 2.5% (v/v) H_2_O in concentrated CF_3_COOH for 4 h. After suspension in Et_2_O, the mixture was centrifuged (5 min, 5000 rpm) in an Eppendorf 5804R centrifuge (Eppendorf, Hamburg, Germany) after which time the supernatant was removed by decanting. The residual solid was washed twice with cold Et_2_O, subjected to centrifugation and dried using a vacuum line overnight. The crude peptide was dissolved in a mixture of MeCN in H_2_O and then purified using RP-HPLC (Thermo Scientific Ultimate 3000 HPLC) and a linear gradient of 12% (v/v) MeCN over 30 min at 3 mL min^−1^ using a Gemini 10 μm NX-C18 110 LC column (Phenomenex, Torrance, CA, USA). Analytical RP-HPLC (Thermo Scientific Ultimate 3000 HPLC) employed a Gemini 5 μm C18 110. The LC column (Phenomenex) was used at a flow rate of 1 mL min^−1^ with a gradient of H_2_O + 0.1% (v/v) CF_3_COOH and MeCN + 0.1% (v/v) CF_3_COOH from 3% (v/v) MeCN to 100% (v/v) MeCN + 0.1% (v/v) CF_3_COOH over 30 min at 1 mL min^−1^. Analytical spectra were recorded at 215 nm.

### LC-MS activity assays

All reagents were purchased from Sigma Aldrich and were of the highest grade available. Ferrous(ii) ammonium sulfate (FAS) solutions were prepared freshly by dissolving FAS to 400 mM in 20 mM HCl with subsequent dilution to 1 mM using deionized water. Fresh 2OG (10 mM) and LAA (50 mM) solutions were prepared by dissolving the solids in deionised water. All enzyme reactions were performed in 384-well plates (Greiner Bio-One) with a final reaction volume of 50 μL. Substrate mixtures contained 50 mM HEPES pH 7.5, LAA (100 μM), FAS (10 μM), 2OG (20 μM) and an H1 isoform peptide (10 μM). Reactions were initiated by the addition of enzyme to a final concentration of 2.0 μM. After 2 h, the reactions were stopped by the addition of 5 μL of 10% (v/v) LC-MS grade formic acid (Fisher Scientific) to a final concentration of 1% (v/v). Product analyses were performed by liquid chromatography-mass spectrometry (LC-MS) using an Agilent 1290 infinity II LC system equipped with an Agilent 1290 multisampler and an Agilent 1290 high speed pump and connected to an Agilent 6550 accurate mass iFunnel quadrupole time of flight (Q-TOF) mass spectrometer. 1 μL of the reaction mixture was injected onto a 1.8 μm × 2.1 × 50 mm ZORBAX RRHD Eclipse Plus C18 column (Agilent). Solvent A consisted of HPLC grade water containing 0.1% (v/v) formic acid and solvent B consisted of MeCN containing 0.1% (v/v) formic acid. Peptides were separated using a stepwise gradient (0 min – 95% solvent A, 1.0 min – 80% solvent A, 3.0 min – 45% solvent A, 4.0 min – 45% solvent A, 5.0 min – 0% solvent A, 6.0 min – 0% solvent A, 7.0 min – 95% solvent A). This was followed by a 3 min post-run with 95% solvent A to re-equilibrate the column. All flow rates were 0.2 mL min^−1^. A blank injection of HPLC grade water was carried out between each sample injection. The mass spectrometer was operated in the positive ion mode with a drying gas temperature of 280 °C, a drying gas flow rate of 13 L min^−1^, a nebulizer pressure of 40 psig, a sheath gas temperature of 350 °C, a sheath gas flow rate of 12 L min^−1^, a capillary voltage of 4000 V, and a nozzle voltage of 1000 V. Data were analysed using Agilent MassHunter Qualitative Analysis (Version B.07.00) software.

### Kinetic analyses

Substrate *K*_m_ values for KDM4 enzymes were determined using a reported formaldehyde dehydrogenase/NAD coupled enzyme assay that quantifies formaldehyde production.^30^ In brief, DNA encoding for wild-type full-length formaldehyde dehydrogenase was synthesised (by GenScript) and cloned into the pET-28B vector. The resultant plasmid was then transformed into the Rosetta strain of *Escherichia coli*. Coupled enzyme-assays were carried out in black polystyrene 384-well non-binding surface microplates (Corning) and kinetic measurements were obtained using a PHERAstar FS plate reader (BMG Labtech) equipped with a 350 nm excitation and 460 nm emission optic module.^30^ All steps were performed in the assay buffer (50 mM HEPES pH 7.5, 0.01% (v/v) Tween-20). Substrate mixtures were prepared freshly and contained FAS (20 μM), LAA (200 μM), 2OG (200 μM), NAD^+^ (500 μM), and H1 peptide (1–50 μM); they were dispensed (25 μL final volume) in triplicate. Assays were initiated by plate reader injection of 25 μL of a mixture of KDM4 (0.8 μM) and formaldehyde dehydrogenase (FDH) (2.0 μM) in assay buffer (50 mM HEPES pH 7.5, 0.01% (v/v) Tween-20). Production of NADH was measured at 60 second intervals over 25 cycles. Kinetic parameters were determined from the reaction rate during the initial linear phase of formaldehyde production, and specific activities were calculated from a formaldehyde standard curve and fitted to the Michaelis–Menten equation using GraphPad Prism Version 5.0 (GraphPad software, La Jolla, CA, USA). Each assay was carried out in triplicate with independently prepared reaction mixtures.

### ^1^H NMR assays

NMR spectra were acquired at 298 K using a Bruker AVIII 700 MHz NMR spectrometer equipped with a TCI helium-cooled CryoProbe. Data were processed using TopSpin v.3.6.2 software. Signal intensities were calibrated relative to TSP, *δ*_H_ 0 ppm, and the chemical shifts (ppm) are relative to the resonance of the solvent, *δ*_H_ 4.7 ppm. Assay mixtures contained KDM4D (10 μM), 2OG (400 μM), H1_20–34_ peptide (400 μM), Fe(ii) ammonium sulfate (100 μM), l-ascorbate (1 mM) and internal standard, 3-(trimethylsilyl)-2,2,3,3-tetradeuteropropionic acid (TSP, 800 μM) in 50 mM Tris-d11, pH 7.5. Aqueous stocks of 2OG, Fe(ii) ammonium sulfate and l-ascorbate were prepared fresh on the day of testing. Samples were transferred to 3-mm diameter MATCH NMR tubes (CortecNet) and monitored by ^1^H excitation sculpting with baseline optimisation using 16 transients. The time lapse between sample mixing and data acquisition was 6–7 min. The spectra were acquired every 2 min over 60 min. Starting material and product concentrations were quantified relative to the TSP concentration. Samples from two independent experiments were analysed by NMR spectroscopy.

## Author contributions

J. M. and C. J. S. conceived and supervised the project. V. A. T. synthesised histone peptides. V. A. T. and A. T. carried out enzyme assays. E. S. produced enzymes. S. K. carried out NMR assays. V. A. T., C. J. S. and J. M. wrote the manuscript with help from other authors.

## Conflicts of interest

There are no conflicts of interest.

## Supplementary Material

CB-006-D5CB00083A-s001

## Data Availability

The data supporting this article have been included as part of the SI. Peptide characterisation, enzyme assays, and NMR spectroscopy. See DOI: https://doi.org/10.1039/d5cb00083a
