# Supplementary material for: Investigating the N-terminal linker histone H1 subtypes as substrates for JmjC lysine demethylases
Source: RSC Chem Biol. 2025 Jul 28;6(10):1607–15. doi: 10.1039/d5cb00083a (PMC12363342; doi:10.1039/d5cb00083a)
Supplement: CB-006-D5CB00083A-s001 [file CB-006-D5CB00083A-s001.pdf]

Supplementary Information (SI) for RSC Chemical Biology.
This journal is © The Royal Society of Chemistry 2025

Supplementary Information

Investigating the N-terminal linker histone H1 subtypes as substrates for JmjC lysine demethylases

Vildan A. Türkmen,[a] Anthony Tumber,[b] Eidarus Salah,[b] Samanpreet Kaur,[b] Christopher J. Schofield*[b] and Jasmin Mecinović*[a]

[a] Department of Physics, Chemistry and Pharmacy, University of Southern Denmark, Campusvej 55, 5230 Odense, Denmark

[b] Department of Chemistry and the Ineos Oxford Institute for Antimicrobial Research, Chemistry Research Laboratory, University of Oxford, 12 Mansfield Road, OX1 3TA Oxford, United Kingdom

Table S1. Characterisation of N^{ε}-methylated H1 somatic peptides (20-mer) used in this study. All peptides were prepared with C-terminal amides. See Experimental Details for synthetic method. The commercially available peptides are marked with *.

Entry	Peptide	Sequence	Sequence	Formula	Monoisotopic Mass (Da)	Purity
**1**	H3K9me	ARTKQTAR**Kme**STGGKA	1-15	$C_{64}H_{120}N_{26}O_{20}$	1572.91	97.6
**2**	H3K9me2	ARTKQTAR**Kme2**STGGKA	1-15	$C_{65}H_{122}N_{26}O_{20}$	1586.93	>99
**3**	H3K9me3	ARTKQTAR**Kme3**STGGKA	1-15	$C_{66}H_{124}N_{26}O_{20}^{+}$	1600.95	*
**4**	H3K36me	SAPATGGV**Kme**KPHRYRPGTVAL	28-48	$C_{97}H_{162}N_{32}O_{25}$	2175.24	>99
**5**	H3K36me2	SAPATGGV**Kme2**KPHRYRPGTVAL	28-48	$C_{98}H_{164}N_{32}O_{25}$	2189.25	*
**6**	H3K36me3	SAPATGGV**Kme3**KPHRYRPGTVAL	28-48	$C_{99}H_{166}N_{32}O_{25}^{+}$	2203.27	*
**7**	H1.2K25me	KKKAA**Kme**KAGGTPRKA	20-34	$C_{68}H_{129}N_{25}O_{16}$	1552.91	97.3
**8**	H1.2K25me2	KKKAA**Kme2**KAGGTPRKA	20-34	$C_{69}H_{131}N_{25}O_{16}$	1566.02	>99
**9**	H1.2K25me3	KKKAA**Kme3**KAGGTPRKA	20-34	$C_{70}H_{133}N_{25}O_{16}^{+}$	1580.96	98.7
**10**	H1.2K26me	KKKAAK**Kme**AGGTPRKA	20-34	$C_{68}H_{129}N_{25}O_{16}$	1552.91	97.4
**11**	H1.2K26me2	KKKAAK**Kme2**AGGTPRKA	20-34	$C_{69}H_{131}N_{25}O_{16}$	1566.02	96.1
**12**	H1.2K26me3	KKKAAK**Kme3**AGGTPRKA	20-34	$C_{70}H_{133}N_{25}O_{16}^{+}$	1580.96	>99
**13**	H1.3K24me	KKKA**Kme**KAGATAGKRK	20-34	$C_{69}H_{134}N_{26}O_{16}$	1583.97	98.6
**14**	H1.3K24me2	KKKA**Kme2**KAGATAGKRK	20-34	$C_{70}H_{136}N_{26}O_{16}$	1597.99	98.1
**15**	H1.3K24me3	KKKA**Kme3**KAGATAGKRK	20-34	$C_{71}H_{138}N_{26}O_{26}^{+}$	1512.02	96.2
**16**	H1.3K25me	KKKAK**Kme**AGATAGKRK	20-34	$C_{69}H_{134}N_{26}O_{16}$	1583.97	>99
**17**	H1.3K25me2	KKKAK**Kme2**AGATAGKRK	20-34	$C_{70}H_{136}N_{26}O_{16}$	1597.99	>99
**18**	H1.3K25me3	KKKAK**Kme3**AGATAGKRK	20-34	$C_{71}H_{138}N_{26}O_{26}^{+}$	1512.02	>99
**19**	H1.4K25me	KKKAR**Kme**SAGAAKRKA	20-34	$C_{69}H_{134}N_{28}O_{16}$	1611.05	>99
**20**	H1.4K25me2	KKKAR**Kme2**SAGAAKRKA	20-34	$C_{70}H_{136}N_{28}O_{16}$	1626.05	>99
**21**	H1.4K25me3	KKKAR**Kme3**SAGAAKRKA	20-34	$C_{71}H_{138}N_{28}O_{16}^{+}$	1639.08	90.4
**22**	H1.4K25me3	EKTPVKKKAR**Kme3**SAGAAKRKA	15-34	$C_{96}H_{180}N_{34}O_{24}^{+}$	2194.67	>99
**23**	H1.5K25me	KKKAT**Kme**KAAGAGAAK	20-34	$C_{63}H_{120}N_{22}O_{16}$	1441.76	>99
**24**	H1.5K25me2	KKKAT**Kme2**KAAGAGAAK	20-34	$C_{64}H_{122}N_{22}O_{16}$	1455.79	>99
**25**	H1.5K25me3	KKKAT**Kme3**KAAGAGAAK	20-34	$C_{65}H_{124}N_{22}O_{16}^{+}$	1469.82	>99
**26**	H1.5K26me	KKKATK**Kme**AAGAGAAK	20-34	$C_{63}H_{120}N_{22}O_{16}$	1441.76	>99
**27**	H1.5K26me2	KKKATK**Kme2**AAGAGAAK	20-34	$C_{64}H_{122}N_{22}O_{16}$	1455.79	96.1
**28**	H1.5K26me3	KKKATK**Kme3**AAGAGAAK	20-34	$C_{65}H_{124}N_{22}O_{16}^{+}$	1469.82	>99

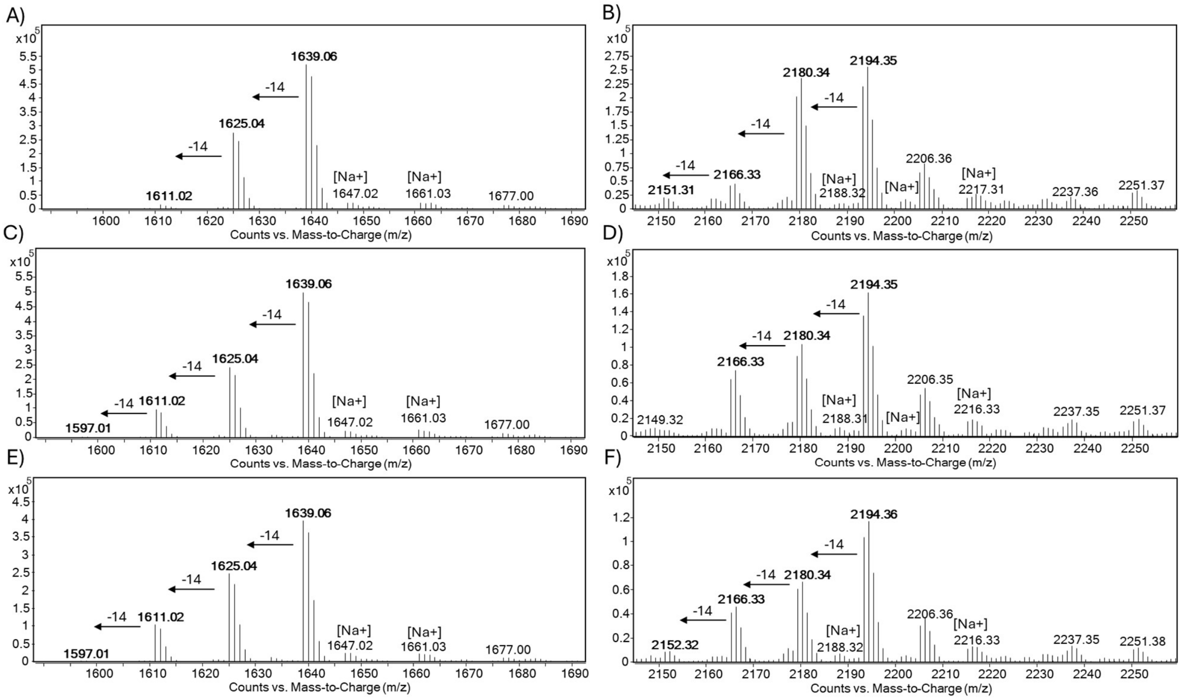

Figure S1. LC-MS spectra for N-terminal demethylations of H1.4K25me3 peptides corresponding to residues: A) H1.4$_{20\text{-}34}$ by KDM4A, B) H1.4$_{15\text{-}34}$ by KDM4A, C) H1.4$_{20\text{-}34}$ by KDM4D, D) H1.4$_{15\text{-}34}$ by KDM4D, E) H1.4$_{20\text{-}34}$ by KDM4E and F) H1.4$_{15\text{-}34}$ by KDM4E.

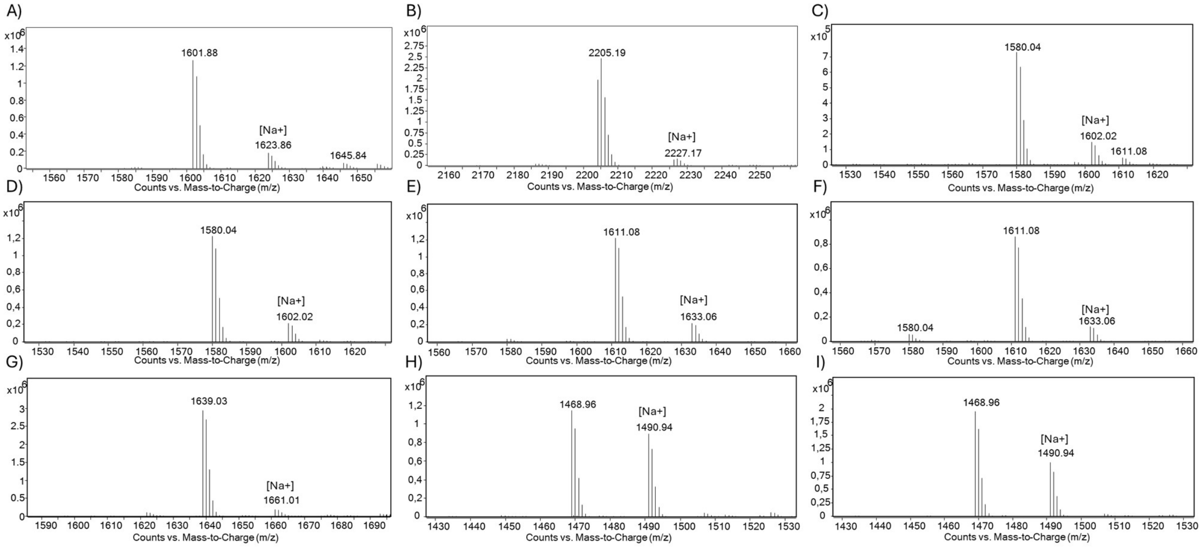

Figure S2. LC-MS spectra for KDM3A-catalysed lysine demethylations of: A) H3K9me3, B) H3K36me3, C) H1.2K25me3, D) H1.2K26me3, E) H1.3K24me3, F) H1.3K25me3, G) H1.4K25me3, H) H1.5K25me3 and I) H1.5K26me3.

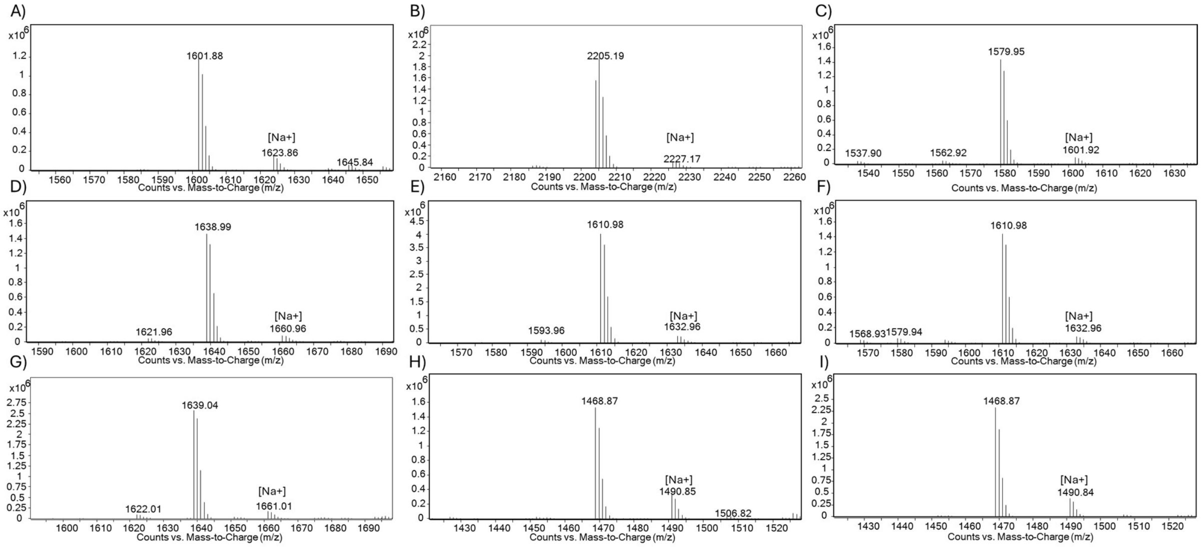

Figure S3. LC-MS spectra for KDM3B-catalysed lysine demethylationss of: A) H3K9me3, B) H3K36me3, C) H1.2K25me3, D) H1.2K26me3, E) H1.3K24me3, F) H1.3K25me3, G) H1.4K25me3, H) H1.5K25me3 and I) H1.5K26me3.

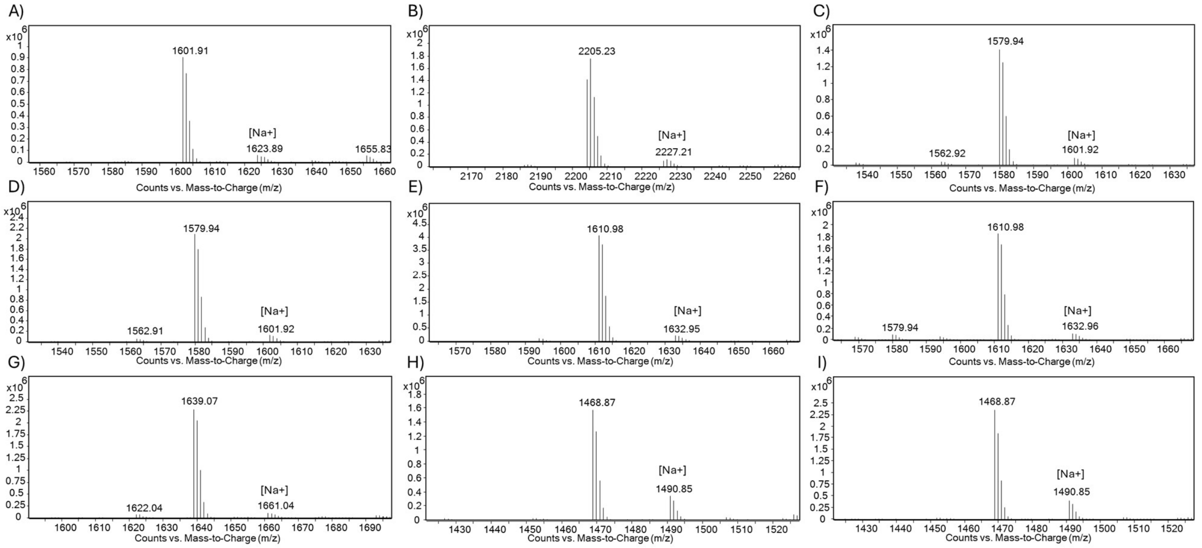

Figure S4. LC-MS spectra for KDM3C-catalysed lysine demethylations of: A) H3K9me3, B) H3K36me3, C) H1.2K25me3, D) H1.2K26me3, E) H1.3K24me3, F) H1.3K25me3, G) H1.4K25me3, H) H1.5K25me3 and I) H1.5K26me3.

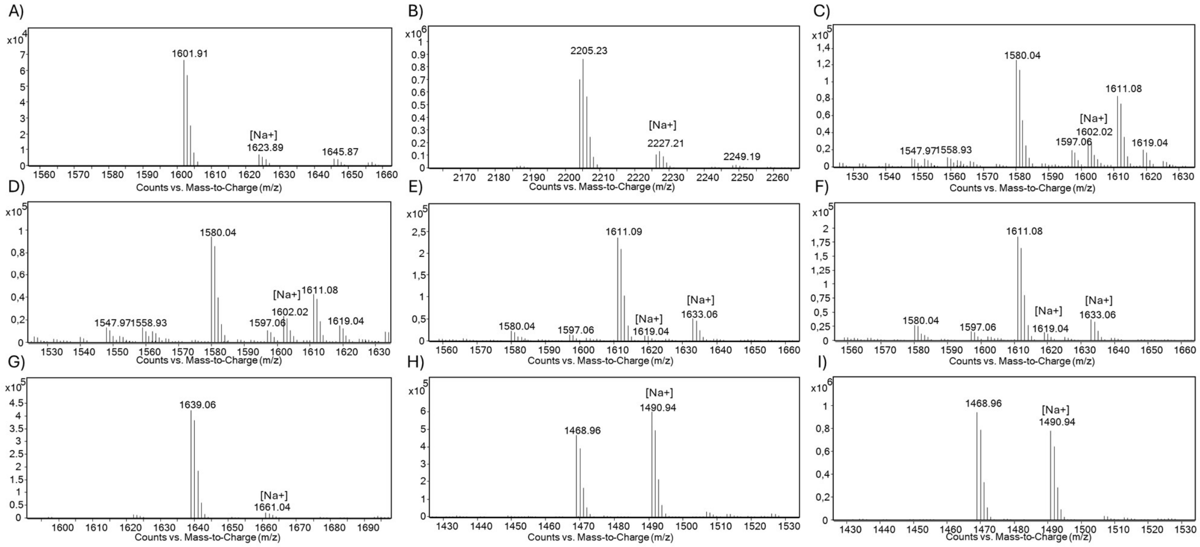

Figure S5. LC-MS spectra for KDM5D-catalysed lysine demethylations of: A) H3K9me3, B) H3K36me3, C) H1.2K25me3, D) H1.2K26me3, E) H1.3K24me3, F) H1.3K25me3, G) H1.4K25me3, H) H1.5K25me3 and I) H1.5K26me3.

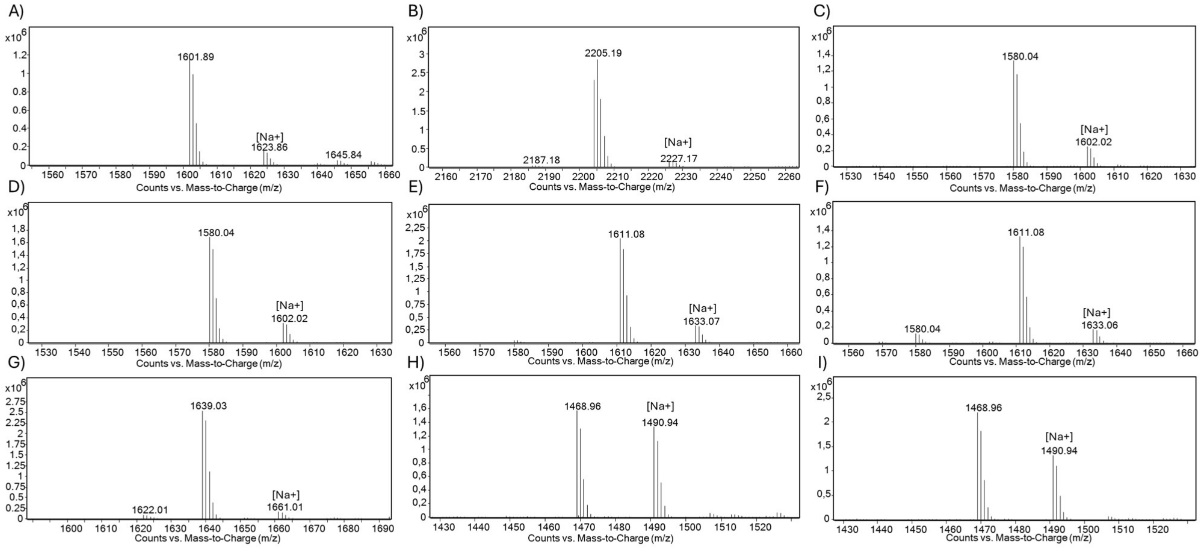

Figure S6. LC-MS spectra for KDM6B-catalysed lysine demethylations of: A) H3K9me3, B) H3K36me3, C) H1.2K25me3, D) H1.2K26me3, E) H1.3K24me3, F) H1.3K25me3, G) H1.4K25me3, H) H1.5K25me3 and I) H1.5K26me3.

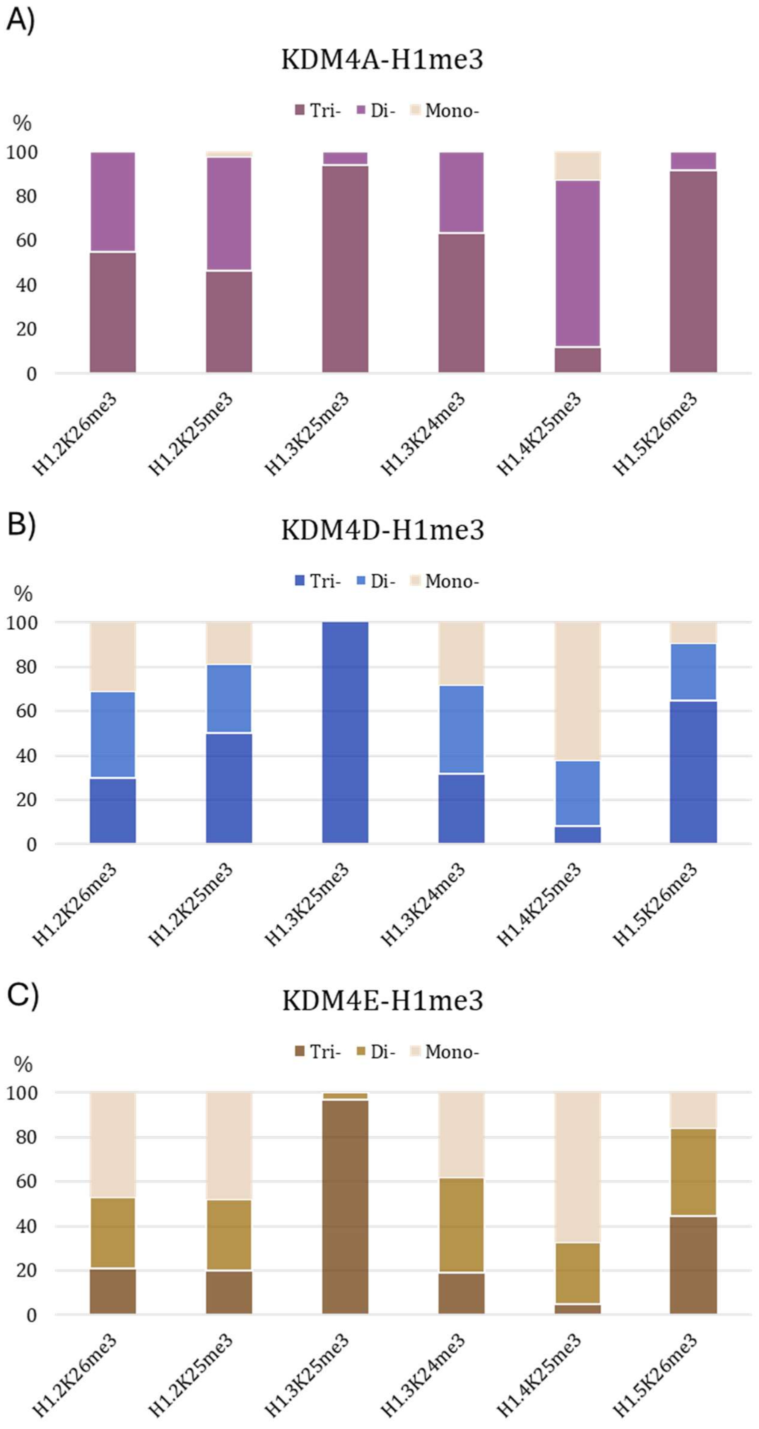

Figure S7. KDM activity summary for trimethylated H1 peptides in the presence of: A) KDM4A, B) KDM4D, and C) KDM4E, shown as tri-, di- and mono-methylated peptides observed after incubation with the specified enzymes. H1.5K25me3 is not included as this peptide did not exhibit any activity. H1.3K25me3 showed minimal conversion.

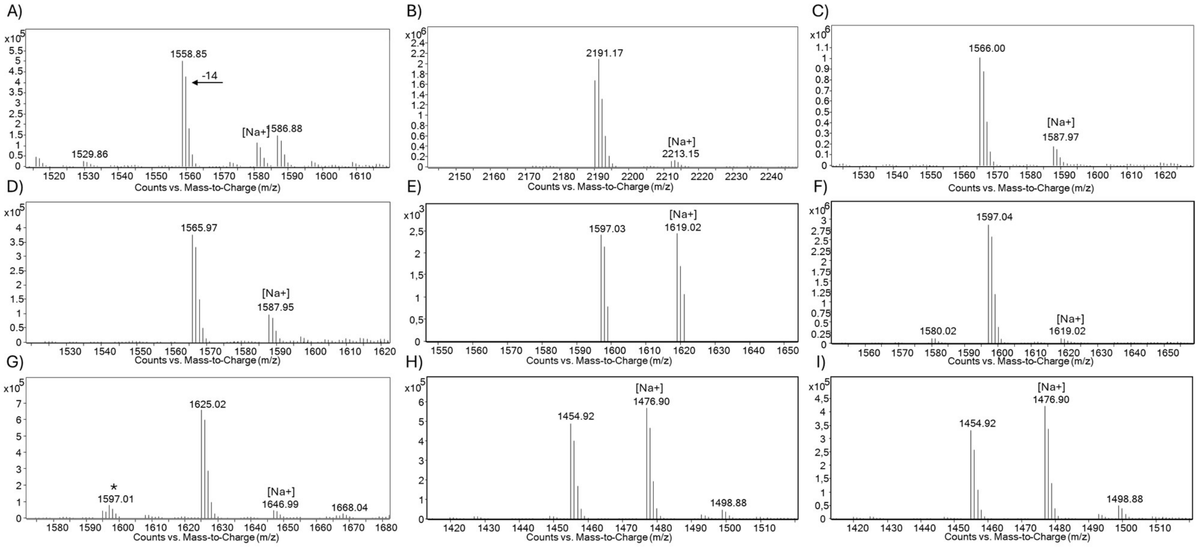

Figure S8. LC-MS spectra for KDM3A-catalysed lysine demethylations of: A) H3K9me2, B) H3K36me2, C) H1.2K25me2, D) H1.2K26me2, E) H1.3K24me2, F) H1.3K25me2, G) H1.4K25me2, H) H1.5K25me2 and I) H1.5K26me2.

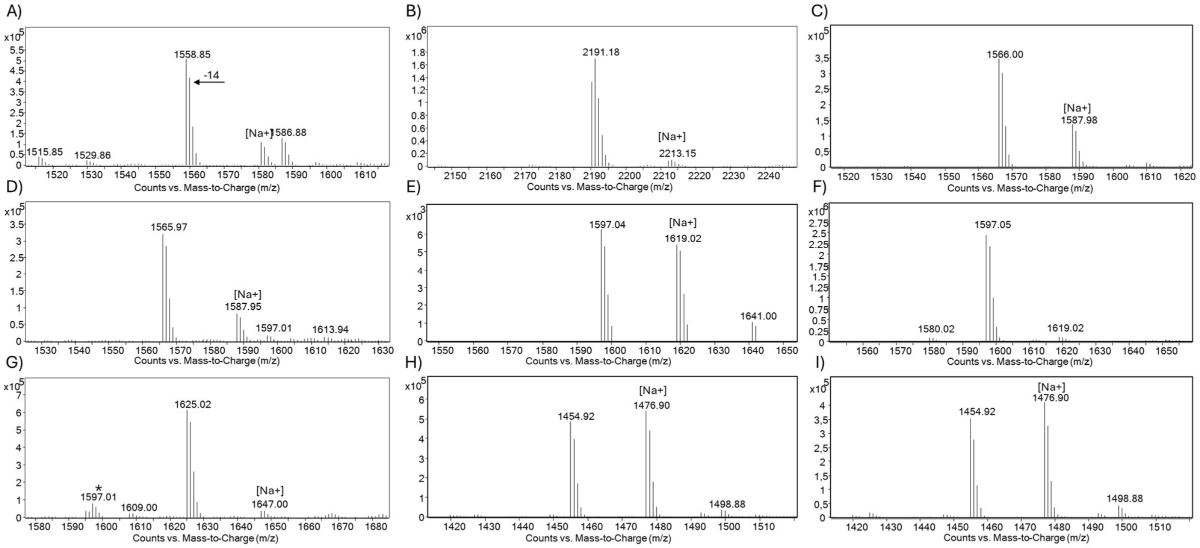

Figure S9. LC-MS spectra for KDM3B-catalysed lysine demethylations of: A) H3K9me2, B) H3K36me2, C) H1.2K25me2, D) H1.2K26me2, E) H1.3K24me2, F) H1.3K25me2, G) H1.4K25me2, H) H1.5K25me2 and I) H1.5K26me2.

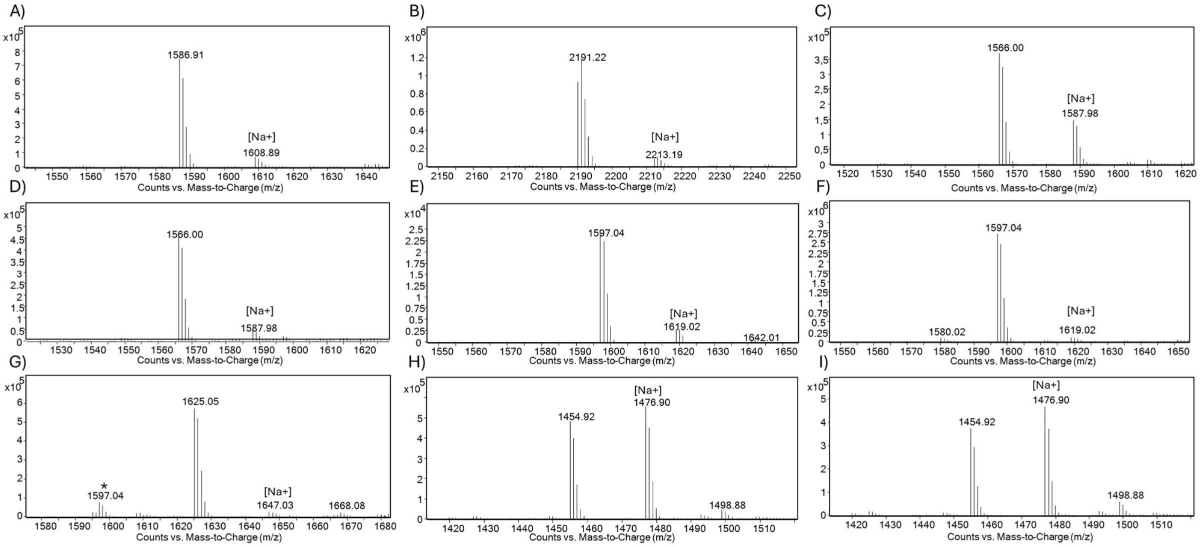

Figure S10. LC-MS spectra for KDM3C-catalysed lysine demethylations of: A) H3K9me2, B) H3K36me2, C) H1.2K25me2, D) H1.2K26me2, E) H1.3K24me2, F) H1.3K25me2, G) H1.4K25me2, H) H1.5K25me2 and I) H1.5K26me2.

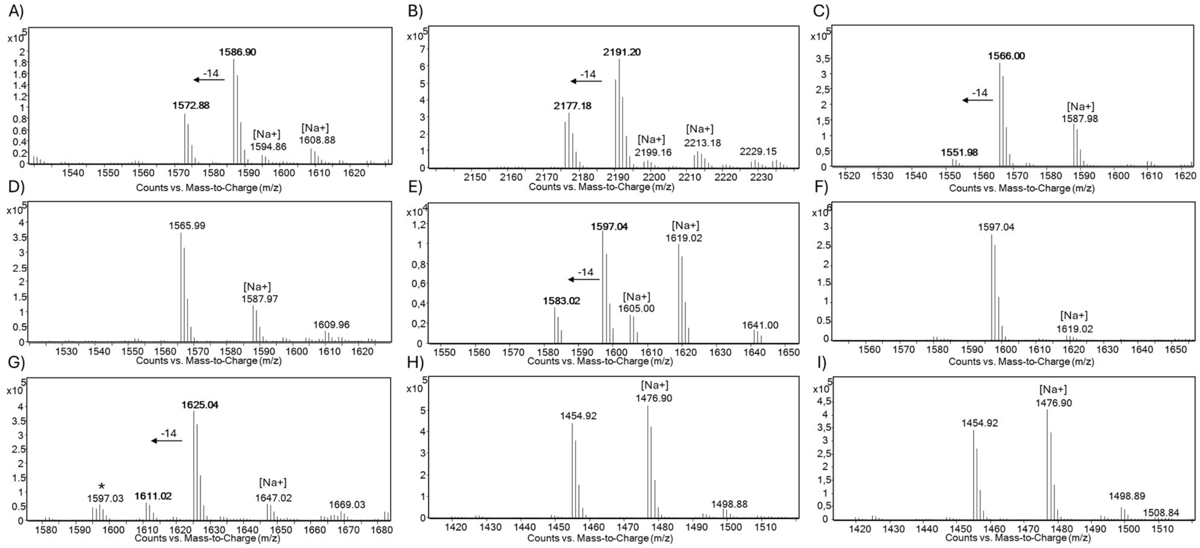

Figure S11. LC-MS spectra for KDM4A-catalysed lysine demethylations of: A) H3K9me2, B) H3K36me2, C) H1.2K25me2, D) H1.2K26me2, E) H1.3K24me2, F) H1.3K25me2, G) H1.4K25me2, H) H1.5K25me2 and I) H1.5K26me2.

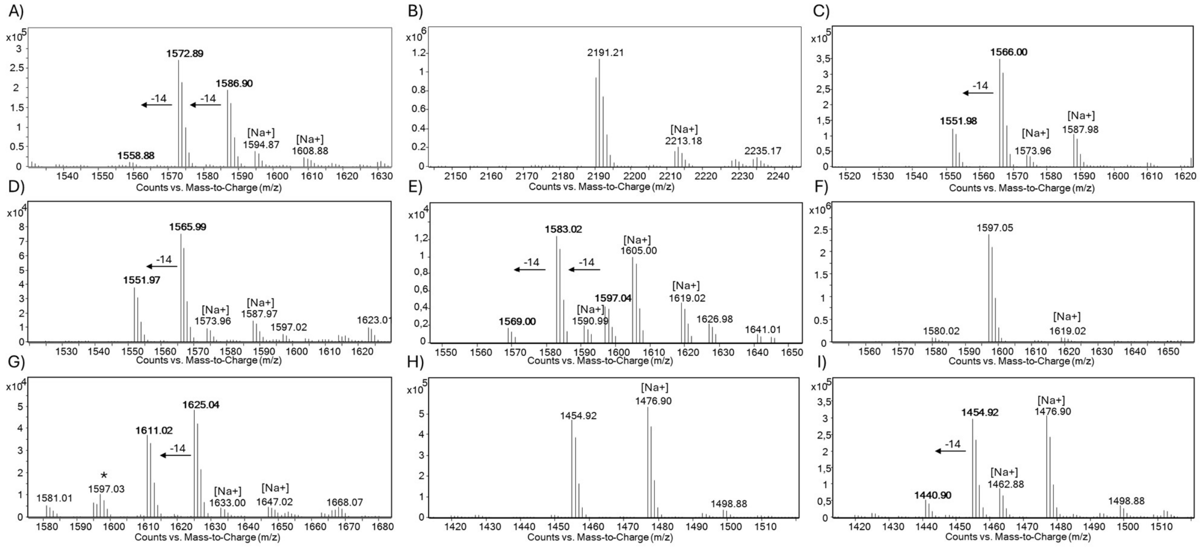

Figure S12. LC-MS spectra for KDM4D-catalysed lysine demethylations of: A) H3K9me2, B) H3K36me2, C) H1.2K25me2, D) H1.2K26me2, E) H1.3K24me2, F) H1.3K25me2, G) H1.4K25me2, H) H1.5K25me2 and I) H1.5K26me2.

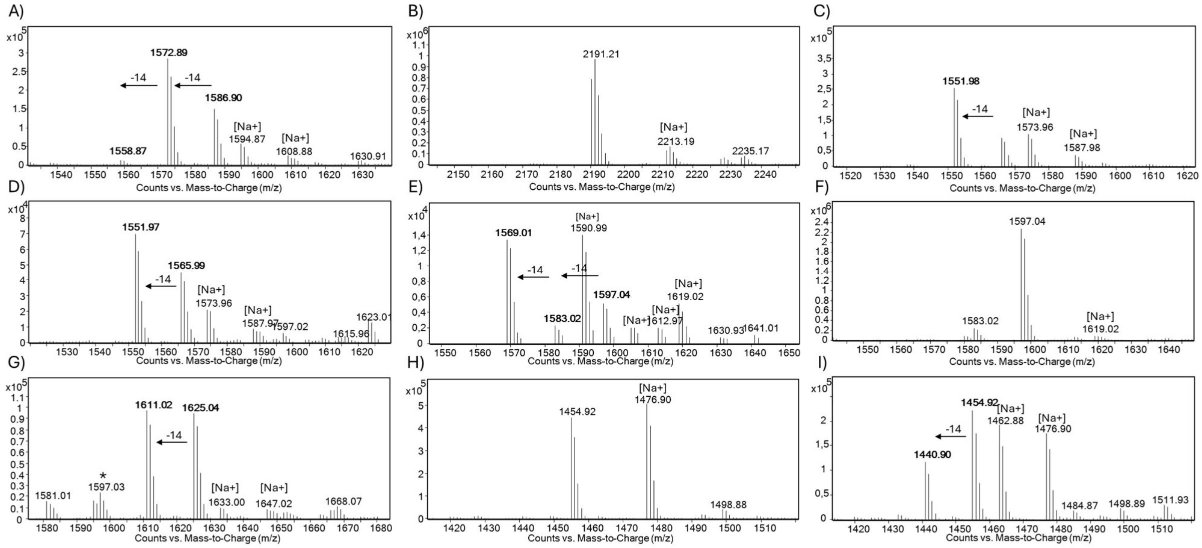

Figure S13. LC-MS spectra for KDM4E-catalysed lysine demethylations of: A) H3K9me2, B) H3K36me2, C) H1.2K25me2, D) H1.2K26me2, E) H1.3K24me2, F) H1.3K25me2, G) H1.4K25me2, H) H1.5K25me2 and I) H1.5K26me2.

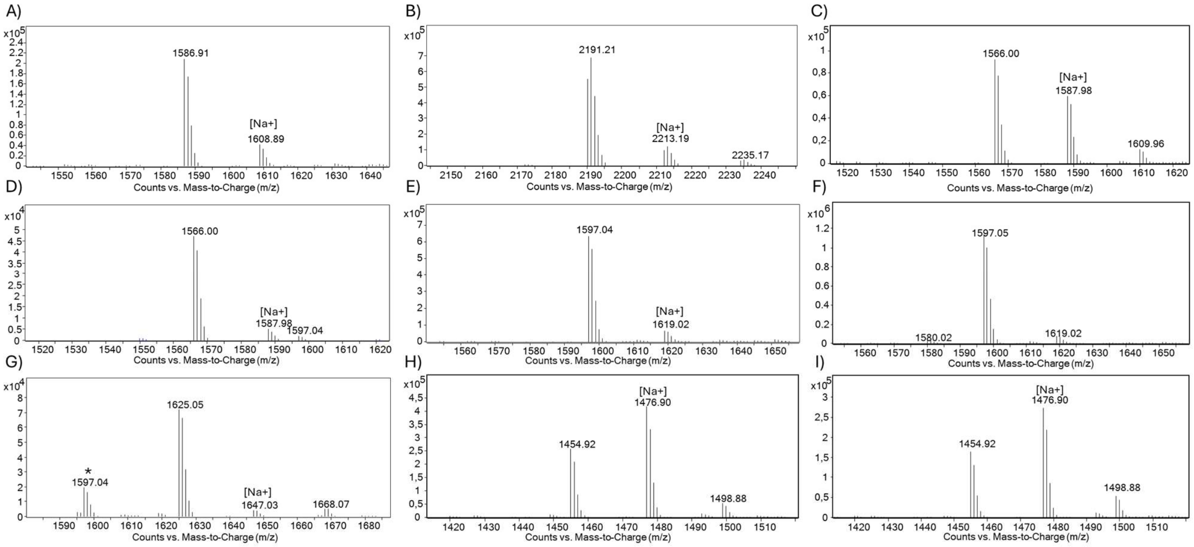

Figure S14. LC-MS spectra for KDM5D-catalysed lysine demethylations of: A) H3K9me2, B) H3K36me2, C) H1.2K25me2, D) H1.2K26me2, E) H1.3K24me2, F) H1.3K25me2, G) H1.4K25me2, H) H1.5K25me2 and I) H1.5K26me2.

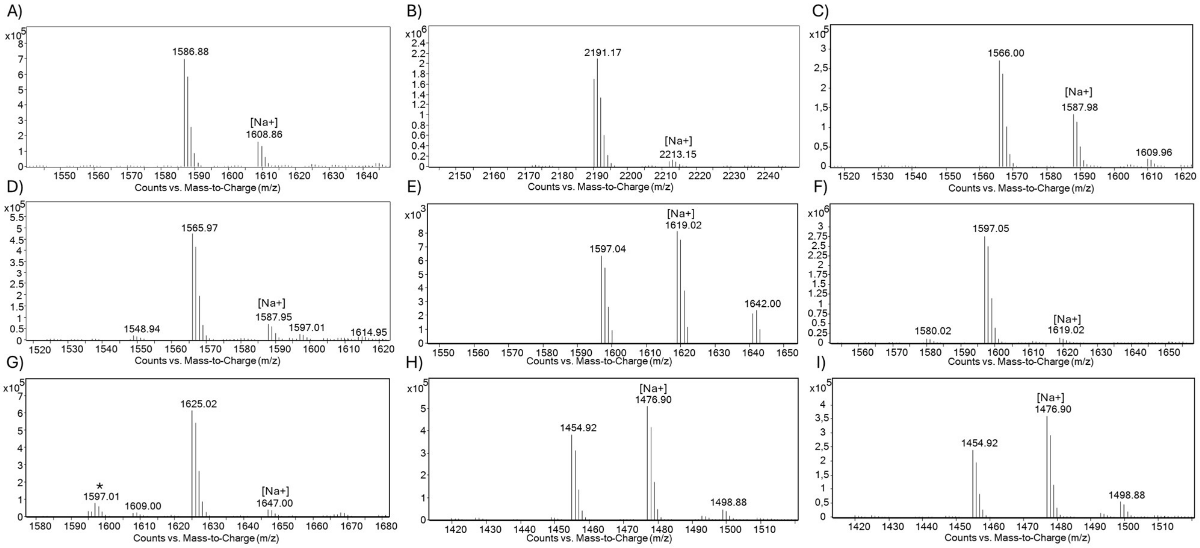

Figure S15. LC-MS spectra for KDM6B-catalysed lysine demethylations of: A) H3K9me2, B) H3K36me2, C) H1.2K25me2, D) H1.2K26me2, E) H1.3K24me2, F) H1.3K25me2, G) H1.4K25me2, H) H1.5K25me2 and I) H1.5K26me2.

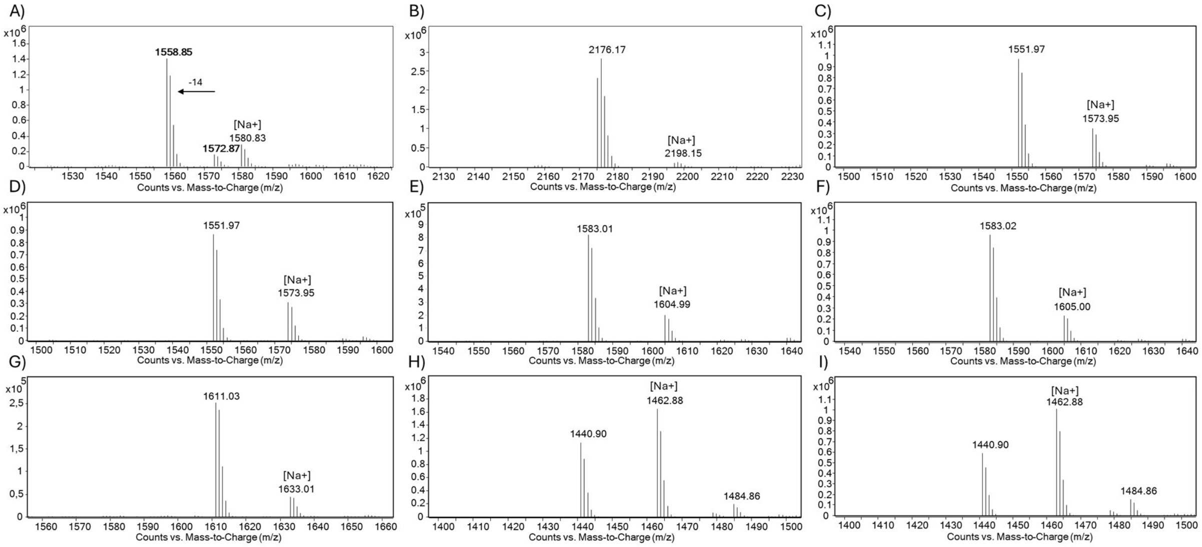

Figure S16. LC-MS spectra for KDM3A-catalysed lysine demethylations of: A) H3K9me, B) H3K36me, C) H1.2K25me, D) H1.2K26me, E) H1.3K24me, F) H1.3K25me, G) H1.4K25me, H) H1.5K25me and I) H1.5K26me.

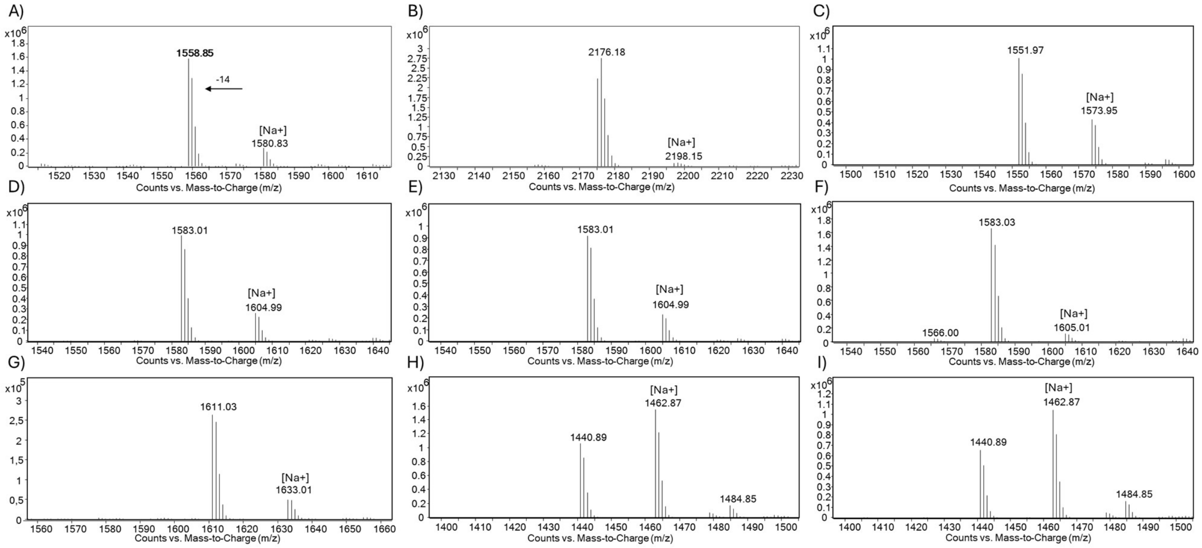

Figure S17. LC-MS spectra for KDM3B-catalysed lysine demethylations of: A) H3K9me, B) H3K36me, C) H1.2K25me, D) H1.2K26me, E) H1.3K24me, F) H1.3K25me, G) H1.4K25me, H) H1.5K25me and I) H1.5K26me.

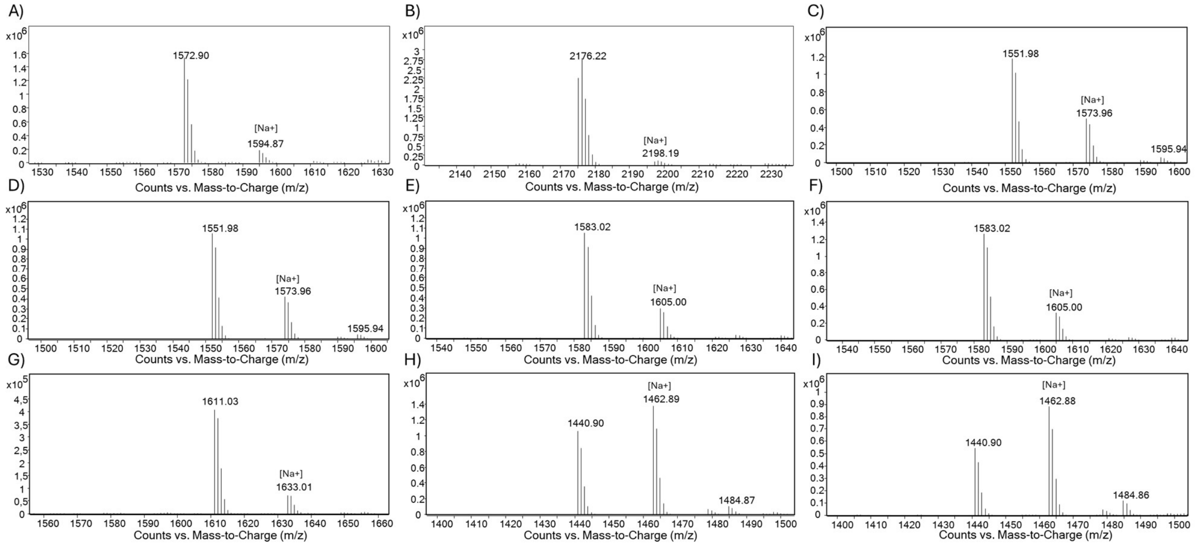

Figure S18. LC-MS spectra for KDM3C-catalysed lysine demethylations of: A) H3K9me, B) H3K36me, C) H1.2K25me, D) H1.2K26me, E) H1.3K24me, F) H1.3K25me, G) H1.4K25me, H) H1.5K25me and I) H1.5K26me.

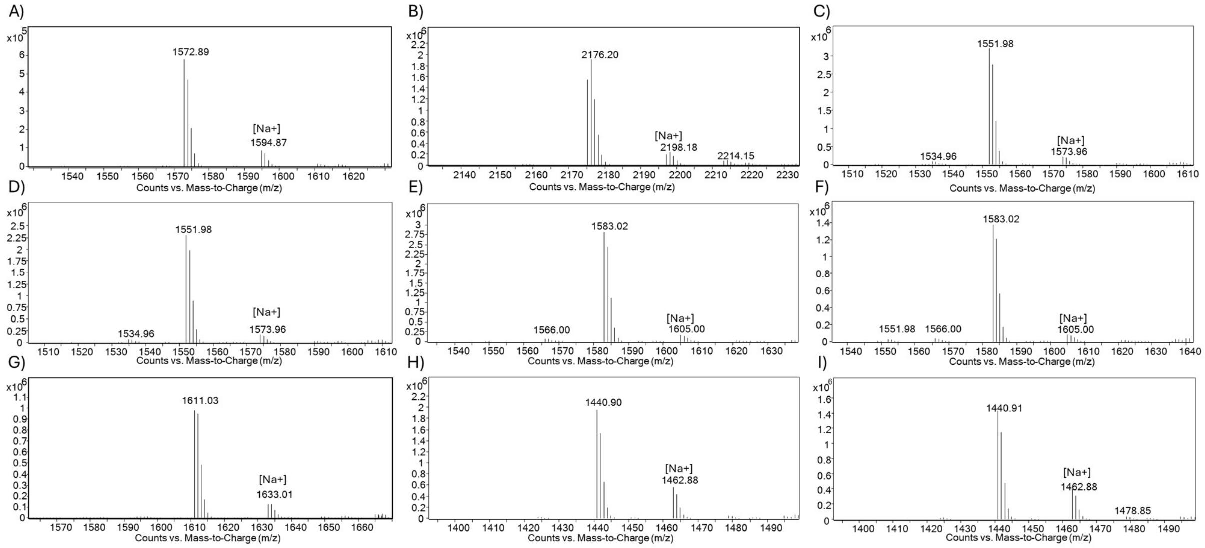

Figure S19. LC-MS spectra for KDM4A-catalysed lysine demethylations of: A) H3K9me, B) H3K36me, C) H1.2K25me, D) H1.2K26me, E) H1.3K24me, F) H1.3K25me, G) H1.4K25me, H) H1.5K25me and I) H1.5K26me.

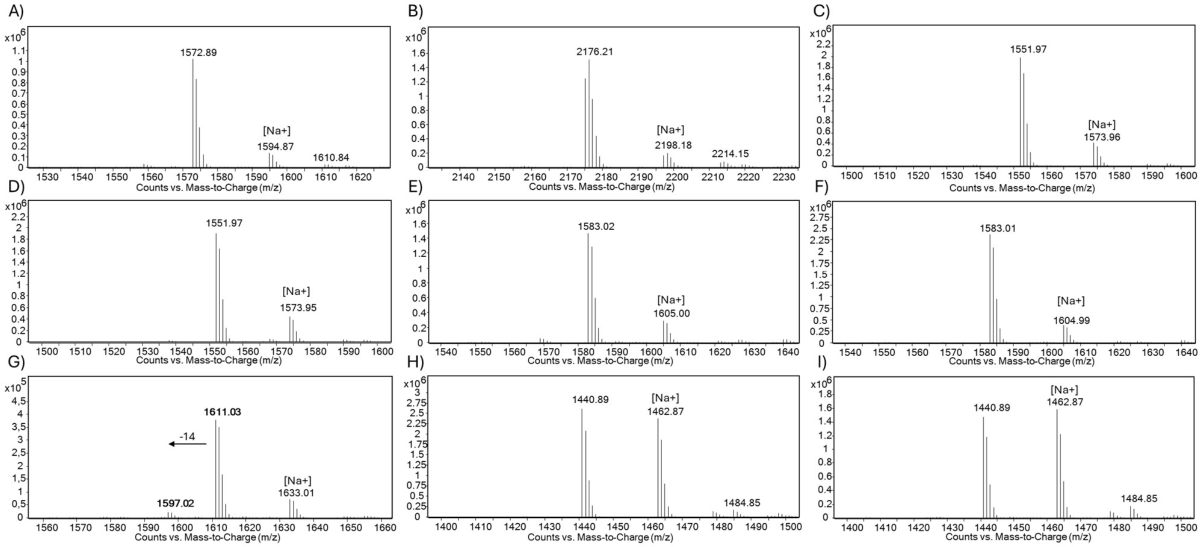

Figure S20. LC-MS spectra for KDM4D-catalysed lysine demethylations of: A) H3K9me, B) H3K36me, C) H1.2K25me, D) H1.2K26me, E) H1.3K24me, F) H1.3K25me, G) H1.4K25me, H) H1.5K25me and I) H1.5K26me.

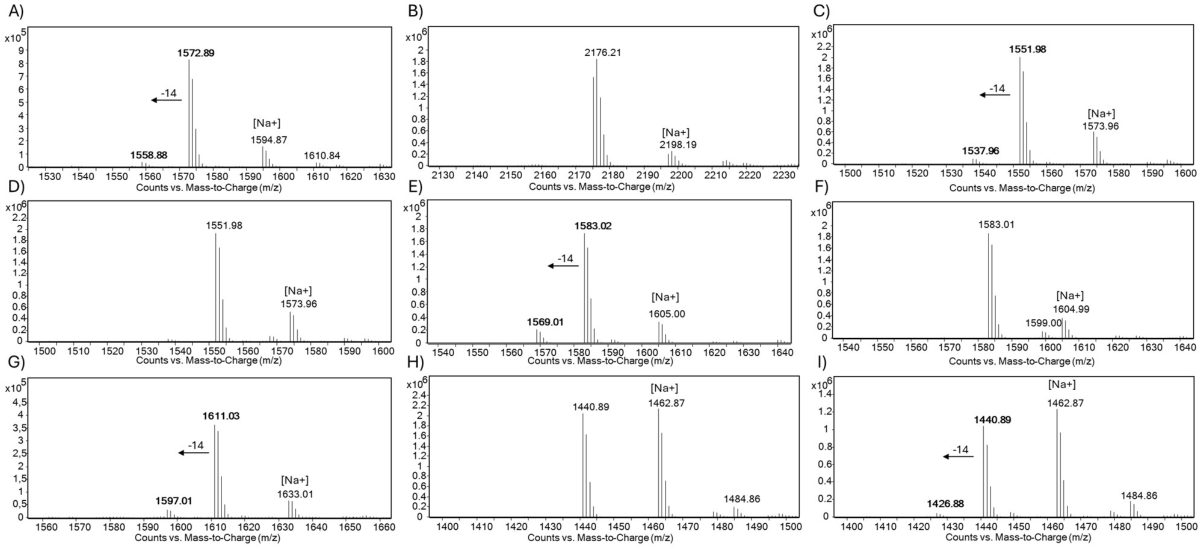

Figure S21. LC-MS spectra for KDM4E-catalysed lysine demethylations of: A) H3K9me, B) H3K36me, C) H1.2K25me, D) H1.2K26me, E) H1.3K24me, F) H1.3K25me, G) H1.4K25me, H) H1.5K25me and I) H1.5K26me.

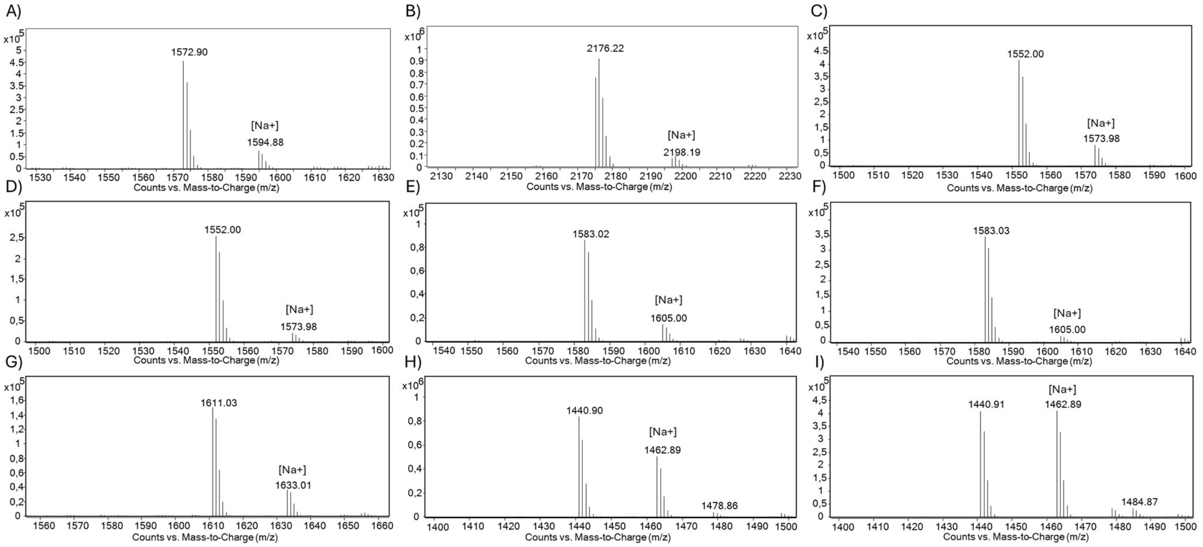

Figure S22. LC-MS spectra for KDM5D-catalysed lysine demethylations of: A) H3K9me, B) H3K36me, C) H1.2K25me, D) H1.2K26me, E) H1.3K24me, F) H1.3K25me, G) H1.4K25me, H) H1.5K25me and I) H1.5K26me.

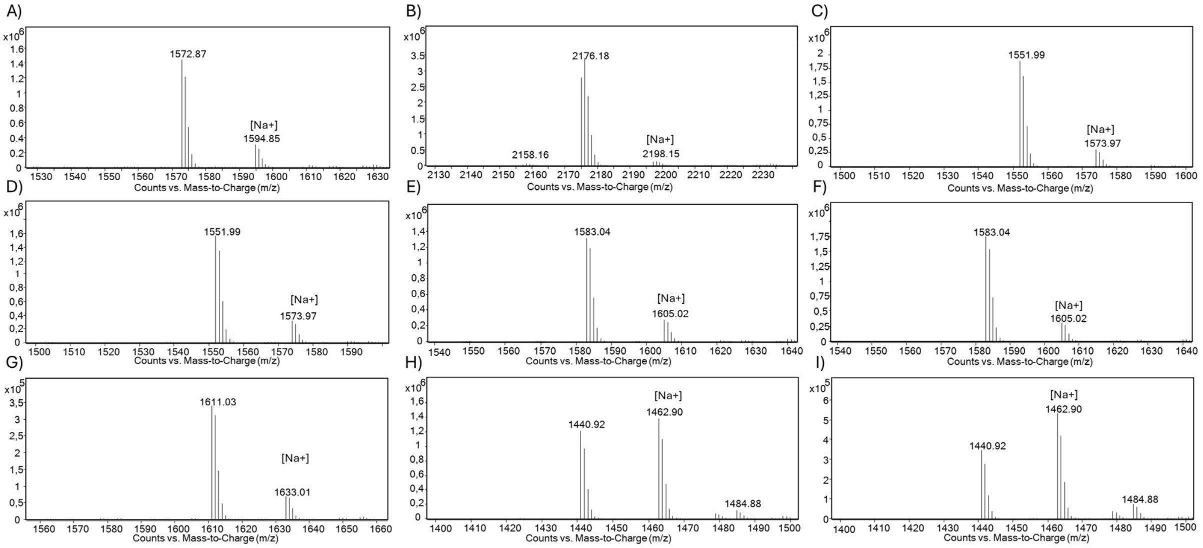

Figure S23. LC-MS spectra for KDM6B-catalysed lysine demethylations of: A) H3K9me, B) H3K36me, C) H1.2K25me, D) H1.2K26me, E) H1.3K24me, F) H1.3K25me, G) H1.4K25me, H) H1.5K25me and I) H1.5K26me.

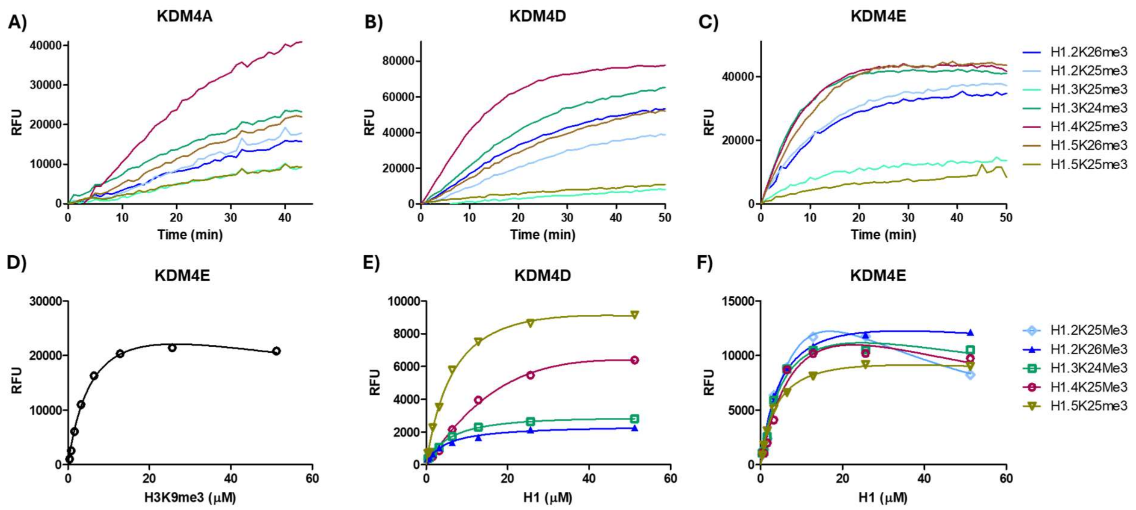

Figure S24. KDM activity determination by the FDH assay for H1 variants (10 μM) in the presence of KDM4 (400 nM). Assay conditions are described in the Experimental Details: Kinetic analyses. Time-course data using A) KDM4A (400 nM), B) KDM4D (400 nM) and KDM4E (400 nM). Kinetics of demethylation of H1 peptides by recombinant human: D) KDM4E (400 nM), E) KDM4D (400 nM) and F) KDM4E (400 nM) measured using the FDH-coupled demethylation assay.[1]

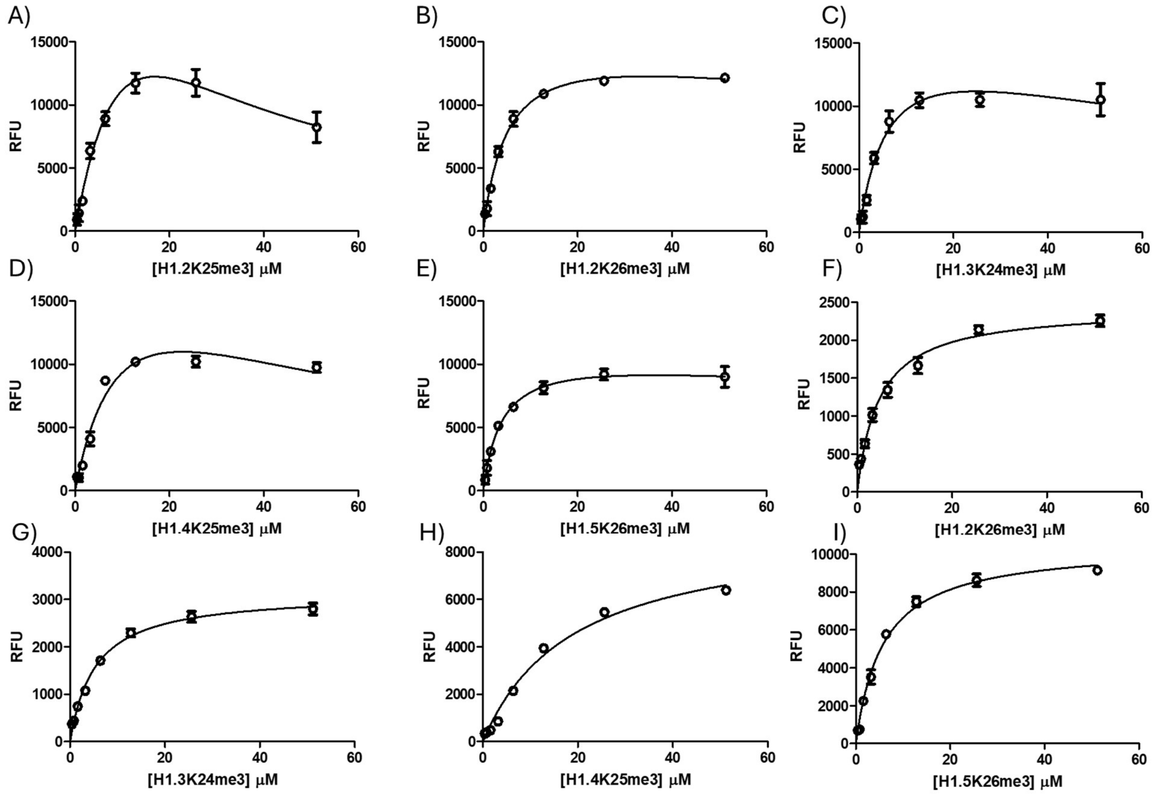

Figure S25. Michaelis-Menten kinetics for H1 peptide demethylation as catalysed by recombinant human (A-E) KDM4E (400 nM) and (F-I) KDM4D (400 nM) measured using a formaldehyde dehydrogenase-coupled demethylation assay.[1] Assay conditions are described in the Experimental Details: Kinetic analyses. Note in the cases of H1.2K25me3 (A) and H1.4K25me3 (D), there is evidence for substrate inhibition as precedented in the case of some other 2OG dependent oxygenases (for recent examples see, e.g.,[2, 3]).

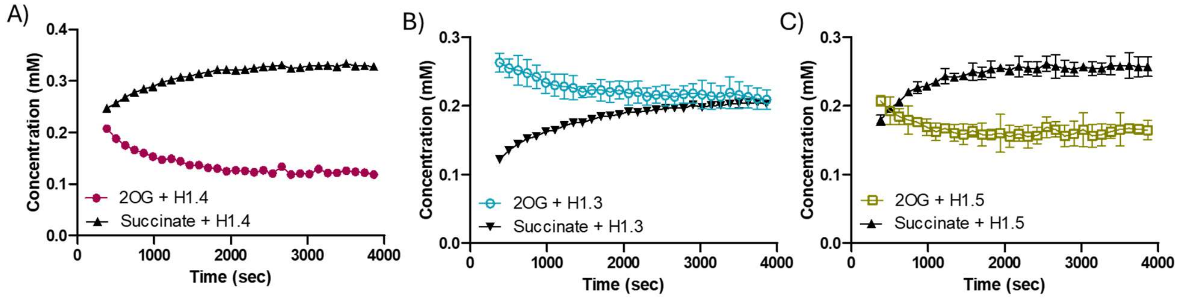

Figure S26. Graphs showing the degree of succinate production and peptide demethylation with: A) H1.4K25me3, B) H1.3K25me3 and C) H1.5K25me3 peptides catalysed by KDM4D as quantified by ^{1}H NMR (700 MHz). Assay conditions are described in the Experimental Details: ^{1}H NMR assays.

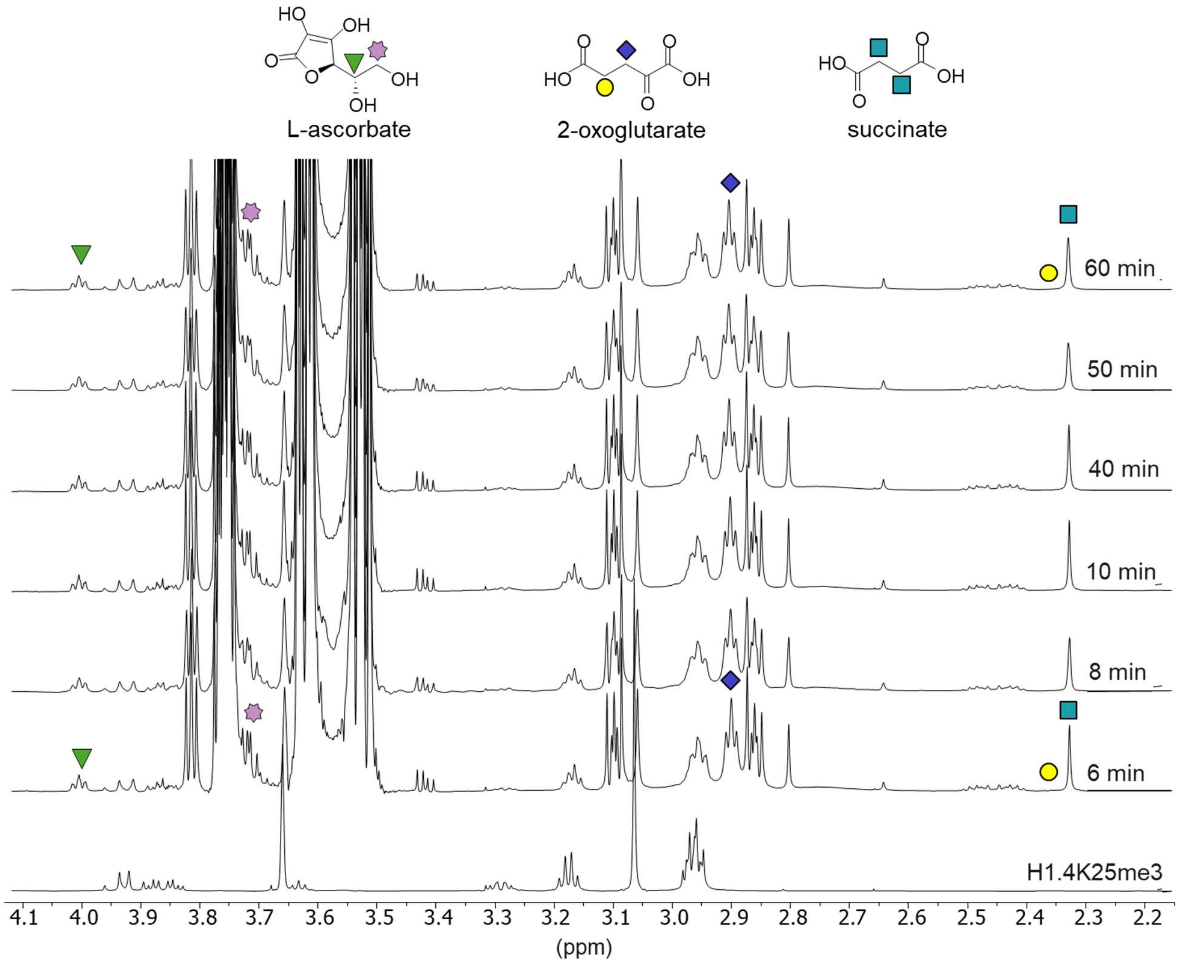

Figure S27. Time-course ^{1}H NMR (700 MHz) for H1.4K25me3 in presence of KDM4D. Complete turnover of 2OG to succinate is observed after 30 min. Assay conditions are described in the Experimental Details: ^{1}H NMR assays.

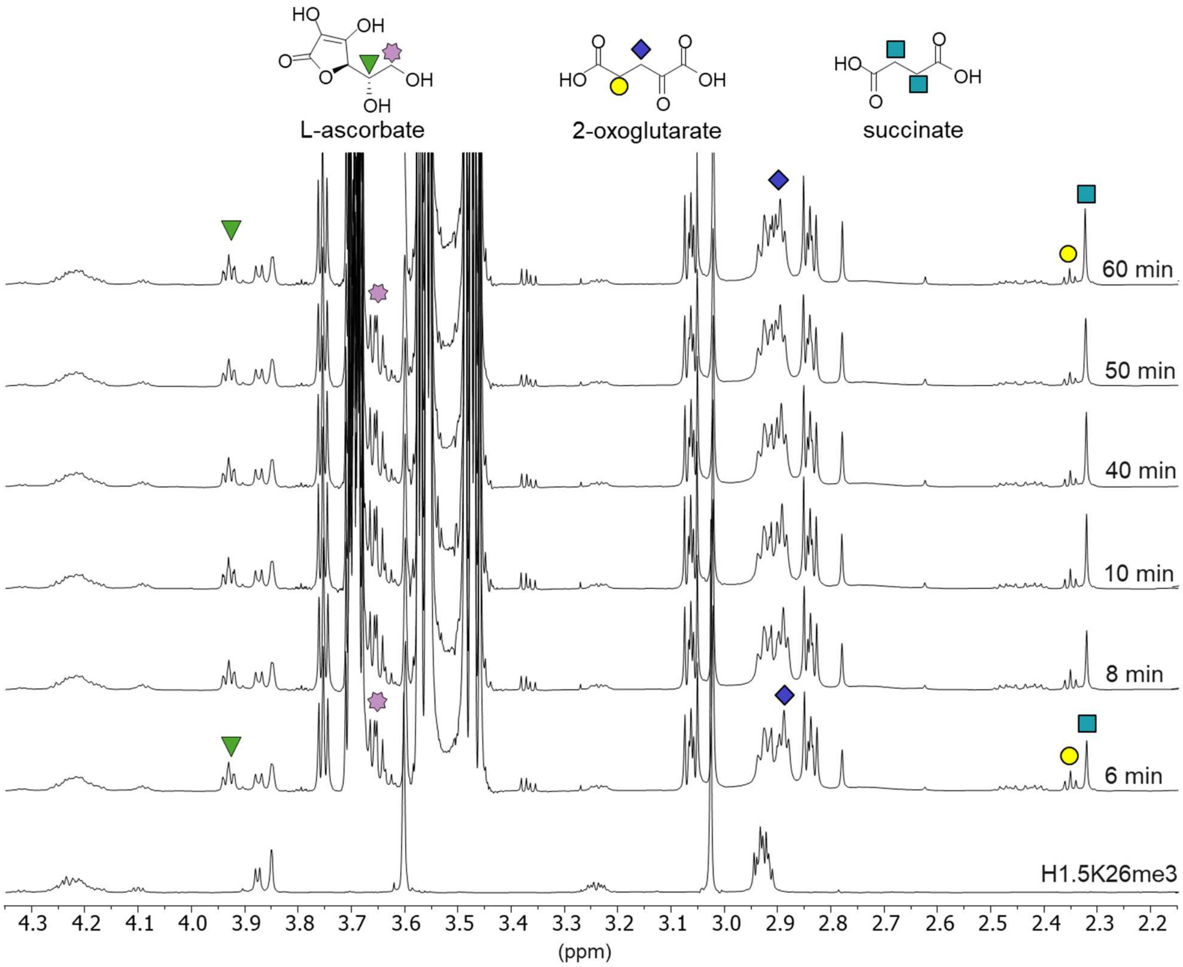

Figure S28. Time-course ^{1}H NMR (700 MHz) for H1.5K26me3 in presence of KDM4D. Assay conditions are described in the Experimental Details: ^{1}H NMR assays.

References

1. N. R. Rose, S. S. Ng, J. Mecinović, B. M. Liénard, S. H. Bello, Z. Sun, M. A. McDonough, U. Oppermann and C. J. Schofield, *J. Med. Chem.*, 2008, **51**, 7053-7056.
2. L. Brewitz, A. Tumber and C. J. Schofield, *J. Biol. Chem.*, 2020, **295**, 7826-7838.
3. T. P. Corner, E. Salah, A. Tumber, L. Brewitz and C. J. Schofield, *RSC Chem. Biol.*, 2025, **6**, 642-656.
